# Green Recycling Supplier Selection of Shared Bicycles: Interval-Valued Pythagorean Fuzzy Hybrid Weighted Methods Based on Self-Confidence Level

**DOI:** 10.3390/ijerph19095024

**Published:** 2022-04-20

**Authors:** Yan Pan, Yanzhe Li, Shouzhen Zeng, Junfang Hu, Kifayat Ullah

**Affiliations:** 1 School of Business, Ningbo University, Ningbo 315211, China; panyan492@163.com (Y.P.); 15857551838@163.com (J.H.); 2 School of Economics and Law, University of Science and Technology Liaoning, Anshan 114051, China; 3 School of Statistics and Mathematics, Zhejiang Gongshang University, Hangzhou 310018, China; 4 Department of Mathematics, Riphah Institute of Computing and Applied Sciences, Riphah International University, Lahore 54000, Pakistan; Kifayat.Khan.Dr@gmail.com

**Keywords:** self-confidence level, interval-valued Pythagorean fuzzy set, hybrid aggregation, MADM, supplier selection, shared bicycle

## Abstract

In the face of practical problems such as the increasing demand for shared bicycles and the number of faulty vehicles which are hard to handle and repair in time, shared bicycles operators tend to outsource recycling services to suppliers. To solve the problem of recycling supplier selection, this paper constructs a novel evaluation index system involving the three traditional dimensions and introduces an interval-valued Pythagorean fuzzy (IVPF) hybrid weighted decision-making model based on the self-confidence level. Subsequently, the self-confidence IVPF hybrid weighted average geometric operator and self-confidence IVPF ordered hybrid weighted average geometric operator are proposed by integrating the self-confidence level of experts, the superiority of the weighted and geometric average rules. The significant merit of the developed operators is that they can incorporate the self-confidence level of the expert as well as effectively combine the characteristics of the weighted and geometric average mechanism. A multi-attribute decision-making (MADM) framework is then constructed by using the proposed aggregation approach. Finally, on the basis of the established evaluation index system, a case concerning the green recycling supplier selection of shared bicycles is applied to display the superiority and practicability of the presented method.

## 1. Introduction

Under the background of building the “beautiful China”, green environmental protection, low-carbon travel, and the construction of ecological civilization have become the direction pursued by the public. The popularity of shared bicycles is the inevitable result of complying with the background of the times and catering to the needs of the public. However, due to the high daily utilization rate of shared bicycles, lack of effective maintenance, non-standard use behavior, and other factors, the number of shared bicycles in stable condition has been seriously reduced, and a large number of faulty vehicles have caused urban environmental pollution to a certain extent. According to reports, it is estimated that the number of bicycles scrapped each year may reach 20 million, equivalent to the configurable steel weight of five aircraft carriers [1]. If the waste steel cannot be practically reused, there exists a failure to utilize resources [2]. However, due to its high recycling cost and low operating profit, most enterprises do not focus on the recycling of abandoned shared bicycles, nor do they take appropriate ways to handle them [3]. Under this circumstance, it is required to select a proper green supplier of shared bicycles for the recycling service, which can not only promote the resource utilization of enterprises but also alleviate the environmental pollution caused by waste steel.

The evaluation index system of supplier selection previously only considered the importance of economic and environmental aspects, and several authors put forward several relevant selection methods on the basis of this index system [4,5,6]. In fact, the sustainable development of an enterprise should cover three dimensions: economy, environment, and society [7,8,9]. Obviously, the green recycling supplier selection of shared bicycles is a typical multiple attribute decision-making (MADM) problem involving various criteria and alternatives, which expresses the inhomogeneity in the following sides. Firstly, although the relevant research on the evaluation index system of green suppliers is relatively mature, few scholars systematically studied the evaluation index system of shared bicycle recycling service suppliers. Secondly, due to the complexity and uncertainty of the shared bicycle recycling operation mechanism, the evaluation information of the considered indicators is usually fuzzy and inaccurate. Third, in the existing literature, researchers rarely consider the confidence level of decision-makers (experts) in evaluation preference, while Liu et al. [10] pointed out that the level of confidence reflects the psychological behavior of decision-makers with different academic and professional backgrounds. Finally, the integration process of various criteria of the scheme has a decisive impact on the final choice in the process of MADM.

Based on the above-mentioned considerations, in this paper, we shall construct a set of evaluation index systems for the selection of shared bicycle green recycling suppliers and use an interval-valued Pythagorean fuzzy set (IVPFS) [11] to measure the uncertain information in the evaluation process. To reflect the familiarity of decision-makers (experts) with the evaluation index, we propose two new interval-valued Pythagorean fuzzy (IVPF) aggregation models based on the self-confidence level, namely, the self-confidence IVPF hybrid weighted average geometric (SC-IVPFHWAG) operator and self-confidence IVPF ordered hybrid weighted average geometric (SC-IVPFOHWAG) operator. These two operators can effectively integrate the self-confidence level of the decision-makers, as well as incorporate the dominant position of the weighted average and geometric average operators, which enhances the reliability and stability of the results. In addition, the influence of parameters on the results is discussed by varying the value of the proposed aggregation operators. Then various comparative experiments are carried out to prove the effectiveness and superiority of the proposed hybrid method.

The remainder of this paper is assigned as follows: Section 2 shows the relevant literature review of green supplier selection and decision-making methods. Section 3 restates several fundamental concepts of the IVPFS. Section 4 introduces two new IVPF hybrid weighted operators based on the self-confidence level. In Section 5, we develop a novel evaluation index system for the selection of shared bicycle green recycling suppliers and outline the construction of a novel framework based on the proposed operators. Section 6 consists of the application of actual cases, sensitivity analysis, and comparative analysis. Finally, Section 7 draws the conclusion.

## 2. Literature Review

### 2.1. Researches on Green Supplier Selection

In terms of green supplier selection, Yan et al. [12] utilized the data envelopment analysis (DEA) method and constructed a novel multi-criteria programming framework, which possesses the capacity to simultaneously assess the management, greenness, and comprehensive efficiency of candidate suppliers. Haeri and Rezaei [5] quoted the best-worst method (BWM) and a fuzzy grey cognitive map and then developed an improved grey relational analysis (GRA). Integrating the BWM and the improved fuzzy technology of order preference by similarity with the ideal solution (TOPSIS), Lo et al. [13] developed a fuzzy multi-target linear programming to handle the selection of green suppliers. Based on the operation rules and comparison laws of interval 2-tuple linguistic variables, Xu et al. [14] explored a group of aggregation methods to implement the proper selection of green suppliers. To coordinate a tripartite sustainable supply chain, Liu et al. [15] proposed a novel approach by combining the interval-valued least-square pre-nucleolus method and the Nash equilibrium strategy. Shi et al. [16] combined the GRA with TOPSIS and implemented the selection of green suppliers under an interval-valued intuitionistic linguistic environment. To choose suitable suppliers and arrange order quantity reasonably, Duan et al. [17] developed a novel integrated model utilizing the alternating queuing method (AQM) and multi-objective production line planning model. Combining BWM with AQM, Liu et al. [18] presented a novel MADM method to carry out the selection of green suppliers. Aiming to mirror the relative relationship among alternatives, Tao et al. [19] proposed a preference relationship-based dynamic group MADM approach adopting the AQM for the green suppliers’ selection. The combinatorial method can easily manage the fuzziness and variability of experts’ subjective assessments, to obtain an optimal green supplier reliably.

However, only a few scholars pay attention to the selection of shared bicycle green-recycling suppliers [2,20]. Due to the characteristics of complex structure, numerous components, large input, and many faulty vehicles, shared bicycles have caused a serious waste of resources and environmental pollution. Compared with other green suppliers, the selection of shared bicycle recycling suppliers has high requirements for the overall operation scale, waste treatment facilities, environmental legal awareness, and renewable resources recovery qualifications. To address the plight of the recycling industry, Liu et al. [21] introduced a dual regulatory system featuring the deposit refund policy and the minimum recovery rate of second-hand products. In light of the recycling of discarded bicycles, Zhang et al. [2] put forward a novel framework to handle the third-party green supplier selection. Regarding the phenomenon of abandoned bicycles parked indiscriminately, Tang and Yang [20] designed a novel IVPF decision-making method to select an appropriate green supplier to carry out the recycling service. Therefore, it is very necessary to carry out the research on the evaluation of green recycling suppliers for shared bicycles in this paper, which can supplement the gap in relevant research.

### 2.2. Fuzzy MADM Method

Recently, fuzzy sets [22,23,24,25,26,27,28,29,30] have been regarded as one of the most practical tools to retain the fuzzy and uncertain features in MADM problems. The Pythagorean fuzzy set (PFS) [30] offers a wider range of applications, is more flexible than the intuitionistic fuzzy set (IFS) [29] when dealing with ambiguity and uncertainty, and is regarded as one of the most appealing and extensively used tools to handle fuzziness. The notion of the PFS is further generalized by dividing the membership and non-membership degrees into interval values, thereby forming the IVPFS [11]. Owing to its superiority in describing uncertain objects and preserving the fuzzy characteristics of evaluation information, the IVPFS can be perceived as an appropriate tool for handling MADM problems with imprecision [11,22,31,32,33,34].

How to integrate IVPF information is regarded as a pivotal discussion point in MADM problems. Garg [11] introduced two aggregation methods, the IVPF weighted average (IVPFWA) and weighted geometric (IVPFWG) operators, and applied them to integrate the IVPF information. Rahman et al. [31] presented an induced IVPF Einstein hybrid weighted averaging aggregation operator and constructed a novel MADM approach. Mu et al. [22] combined the power average rule with Maclaurin symmetric mean method and developed two IVPF power MSM (IVPFPMSM) operators to handle fuzzy decision-making problems. Senapati and Chen [32] formed several IVPF Hamacher hybrid aggregation operators for the selection of emerging software systems. Wang and Li [33] developed a MADM approach based on the hybrid continuous IVPF fuzzy ordered weighted quadratic average operator and applied it to practical cases. Ayyildiz and Gumus [34] utilized the hybrid BMW and Pythagorean fuzzy (PF) analytic hierarchy process (AHP) to calculate the weight coefficient of the criterion in the supply chain performance evaluation. Apparently, most researchers prefer to integrate multiple aggregation operators to develop hybrid weighted methods to manage complex MADM problems. The hybrid weighted method is also preferred by many scholars to address MADM problems under several fuzzy environments, such as intuitionistic fuzzy (IF) environment [35,36,37,38], Q-rung orthopair fuzzy environment [39,40,41,42], and hesitant fuzzy environment [43,44,45].

However, the above-mentioned research fails to consider the confidence of the decision-makers (experts). In recent years, the importance of the self-confidence level of decision-makers has gained increasing attention in MAGDM problems, which can reflect the preference of decision-makers and be conducive to addressing the uncertainty and diversity. For example, Xia et al. [46] proposed two new aggregation methods by combining the evaluation value and self-confidence level, namely the confidence-induced weighted average and confidence-induced weighted geometric operators. Yu [47] presented a class of confidence IF aggregation methods, such as the confidence IF ordered weighted average and confidence IF Einstein weighted average operators, and then applied them to the evaluation of a doctoral dissertation. Zeng et al. [48] introduced two PF confidence aggregation means and applied them to low-carbon supplier selection. Xu et al. [49] proposed four novel aggregation methods considering the self-confidence level: Self-confidence IVPFWA (SC-IVPFWA), self-confidence IVPF ordered weighted average (SC-IVPFOWA), self-confidence IVPFWG (SC-IVPFWG), and self-confidence IVPF ordered weighted geometric (SC-IVPFOWG) operators. The aforementioned studies have proven from theoretical development to empirical applications that aggregation methods based on the self-confidence level can skillfully combine the familiarity and mastery attributes of the decision-makers with the evaluation information, thereby forming the final judgment value, which enhances the scientific validity and dependability of the evaluation results.

Apparently, there exists a large amount of literature on green supplier selection. Most of them mainly focus on the expansion of traditional decision-making methods, and a few studies adopt information aggregation methods to dispose of the selection of green suppliers. However, the research literature on the selection of shared bicycle suppliers is still scarce, and the existing research methods do not take into account the confidence of decision-makers (experts) in the evaluation information. Based on the above considerations, the innovations of this manuscript include: (1) We construct a novel evaluation index system for the selection of shared bicycle green recycling suppliers; (2) We develop a novel hybrid weighted method based on SC-IVPFHWAG and SC-IVPFOHWAG operators. The proposed aggregation method combines the dominant characteristics of weighted average and geometric average aggregation methods and integrates the familiarity of experts with evaluation criteria; (3) The constructed evaluation index system and the novel proposed hybrid method are applied to select the optimal green recycling supplier of shared bicycles.

## 3. Preliminaries

Several basic definitions of the IVPFS are briefly presented in this part.

**Definition** **1.** ([27]) *Let* $D\left(\left[0,1\right]\right)$ *indicate the set of all closed intervals in* $\left[0,1\right]$ *and let Y denote a non-empty finite set. The definition of IVPFS: M has been given as follows:*

(1) $$M=\left\{\left(y,\mu_{M}\left(y\right),\nu_{M}\left(y\right)\right)\left|y\in Y\right.\right\}$$

where $\mu_{M}\left(y\right)$ and $\nu_{M}\left(y\right)$ denote the membership and non-membership intervals of *M*, respectively, with the functions $\mu_{M}\left(y\right)=\left[a,b\right],\nu_{M}\left(y\right)=\left[c,d\right]\subseteq D\left(\left[0,1\right]\right)$ satisfying $b^{2}+d^{2}\leq 1$. $\pi_{M}\left(y\right)=\langle \sqrt{1-b^{2}-d^{2}},\sqrt{1-a^{2}-c^{2}}\rangle$ indicates the hesitation interval, representing the indeterminacy of *M*. For simplicity, let $\psi =\langle \mu_{M}\left(y\right),\nu_{M}\left(y\right)\rangle$ be an IVPF number (IVPFN) that can be described by $\psi =\langle \left[a,b\right],\left[c,d\right]\rangle$.

**Definition** **2.** ([27]) *Let* $\psi_{i}=\langle \left[a_{i},b_{i}\right],\left[c_{i},d_{i}\right]\rangle \left(i=1,2,3\right)$ *be three IVPF numbers (IVPFNs), with* λ>0*. Then,*

(1) $\psi_{1}⊕\psi_{2}=\langle \left[\sqrt{a_{1}^{2}+a_{2}^{2}-a_{1}^{2}a_{2}^{2}},\sqrt{b_{1}^{2}+b_{2}^{2}-b_{1}^{2}b_{2}^{2}}\right],\left[c_{1}c_{2},d_{1}d_{2}\right]\rangle$;
(2) $\psi_{1}⊗\psi_{2}=\langle \left[a_{1}a_{2},b_{1}b_{2}\right],\left[\sqrt{c_{1}^{2}+c_{2}^{2}-c_{1}^{2}c_{2}^{2}},\sqrt{d_{1}^{2}+d_{2}^{2}-d_{1}^{2}d_{2}^{2}}\right]\rangle$;
(3) $\psi_{3}^{\lambda }=\langle \left[a_{3}^{\lambda },b_{3}^{\lambda }\right],\left[\sqrt{1-{\left(1-c_{3}^{2}\right)}^{\lambda }},\sqrt{1-{\left(1-d_{3}^{2}\right)}^{\lambda }}\right]\rangle$;
(4) $\lambda \psi_{3}=\langle \left[\sqrt{1-{\left(1-a_{3}^{2}\right)}^{\lambda }},\sqrt{1-{\left(1-b_{3}^{2}\right)}^{\lambda }}\right],\left[c_{3}^{\lambda },d_{3}^{\lambda }\right]\rangle$.

To measure two IVPFNs, the definitions of score and accuracy functions are introduced as follows:

**Definition** **3.** ([27]) *Let* $\psi =\langle \left[a,b\right],\left[c,d\right]\rangle$ *be an IVPFN. Its score function is introduced below*:

(2) $$S\left(\psi \right)=\frac{1}{2}\left(a^{2}+b^{2}-c^{2}-d^{2}\right)$$

Its accuracy function is stated as follows:

(3) $$H\left(\psi \right)=\frac{1}{2}\left(a^{2}+b^{2}+c^{2}+d^{2}\right)$$

The score function $S\left(\psi \right)$ measures the extent to which the IVPFN belongs to a particular set. For any two IVPFNs, a larger $S\left(\psi \right)$ value corresponds to a larger IVPFN. The accuracy function $H\left(\psi \right)$ addresses the accuracy of the IVPFN information. When two IVPFNs are equal, a larger $H\left(\psi \right)$ means a smaller lag, and thus, the corresponding IVPFN is larger.

**Definition** **4.** ([27]) *Let* $\psi_{1}=\langle \left[a_{1},b_{1}\right],\left[c_{1},d_{1}\right]\rangle ,\psi_{2}=\langle \left[a_{2},b_{2}\right],\left[c_{2},d_{2}\right]\rangle$ *be two IVPFNs.* $S\left(\psi_{1}\right),S\left(\psi_{2}\right)$ *denotes their corresponding score function and* $H\left(\psi_{1}\right),H\left(\psi_{2}\right)$ *denotes their corresponding accuracy function. Then,*

(1) *If*$S\left(\psi_{1}\right)>S\left(\psi_{2}\right)$*, then*$\psi_{1}>\psi_{2}$*;*
(2) *If*$S\left(\psi_{1}\right)<S\left(\psi_{2}\right)$*, then*$\psi_{1}<\psi_{2}$*;*
(3) *If*$S\left(\psi_{1}\right)=S\left(\psi_{2}\right)$*,*

- *If*$H\left(\psi_{1}\right)>H\left(\psi_{2}\right)$*, then*$\psi_{1}>\psi_{2}$*;*
- *If*$H\left(\psi_{1}\right)<H\left(\psi_{2}\right)$*, then*$\psi_{1}<\psi_{2}$*;*
- *If*$H\left(\psi_{1}\right)=H\left(\psi_{2}\right)$*, then*$\psi_{1}=\psi_{2}$.

## 4. Self-Confidence IVPF Hybrid Weighted Operators

It is noted that many existing studies suppose that decision-makers denote a consistent level of knowledge of the attributes representing the estimated objects. However, in realistic conditions, decision-makers often possess diverse social backgrounds, which results in an inconsistent understanding of the assessed objects. Thus, it is important to require decision-makers to provide a self-confidence level that is defined by the degree to which they are confident with the attributes of the assessed objects to make the final evaluation information more referential and reasonable. Under this motivation, Xu et al. [49] integrated the self-confidence level of decision-makers into the evaluation information and defined four self-confidence-weighted operators: the SC-IVPFWA, SC-IVPFOWA, SC-IVPFWG, and SC-IVPFOWG operators. The specific concepts are defined as follows:

**Definition** **5.** ([49]) *Let* $\psi_{i}=\langle \left[a_{i},b_{i}\right],\left[c_{i},d_{i}\right]\rangle \left(i=1,2,\cdots ,n\right)$ *be n IVPFNs, where* $l_{i}$ *means the self-confidence level of* $\psi_{i}$*. The definition of SC-IVPFWA takes the following form:*

(4) $$\begin{array}{l} SC-IVPFWA\left(\left(l_{1},\psi_{1}\right),\left(l_{2},\psi_{2}\right),\cdots ,\left(l_{n},\psi_{n}\right)\right)={⊕}_{i=1}^{n}w_{i}\left(l_{i}\psi_{i}\right)\\ =\langle \left[\sqrt{1-\prod_{i=1}^{n}{\left(1-{\left(a_{i}\right)}^{2}\right)}^{l_{i}w_{i}}},\sqrt{1-\prod_{i=1}^{n}{\left(1-{\left(b_{i}\right)}^{2}\right)}^{l_{i}w_{i}}}\right],\left[\prod_{i=1}^{n}{\left(c_{i}\right)}^{l_{i}w_{i}},\prod_{i=1}^{n}{\left(d_{i}\right)}^{l_{i}w_{i}}\right]\rangle \end{array}$$

where $w_{i}$ is the weight coefficient of $\psi_{i}$ such that $0\leq w_{i}\leq 1$ and $\sum_{i=1}^{n}w_{i}=1$ for i=1,2,⋯,n.

**Definition** **6.** ([49]) *Let* $\psi_{i}=\langle \left[a_{i},b_{i}\right],\left[c_{i},d_{i}\right]\rangle \left(i=1,2,\cdots ,n\right)$ *be n IVPFNs, where* $l_{\delta \left(i\right)}$ *denotes the self-confidence level of* $\psi_{\delta \left(i\right)}$*,* $l_{\delta \left(i\right)}\in \left[0,1\right]$*. The definition of SC-IVPFOWA is as follows:*

(5) $$\begin{array}{l} SC-IVPFOWA\left(\left(l_{1},\psi_{1}\right),\left(l_{2},\psi_{2}\right),\cdots ,\left(l_{n},\psi_{n}\right)\right)={⊕}_{i=1}^{n}w_{i}\left(l_{\delta \left(i\right)}\psi_{\delta \left(i\right)}\right)\\ =\langle \left[\sqrt{1-\prod_{i=1}^{n}{\left(1-{\left(a_{\delta \left(i\right)}\right)}^{2}\right)}^{l_{\delta \left(i\right)}w_{i}}},\sqrt{1-\prod_{i=1}^{n}{\left(1-{\left(b_{\delta \left(i\right)}\right)}^{2}\right)}^{l_{\delta \left(i\right)}w_{i}}}\right],\left[\prod_{i=1}^{n}{\left(c_{\delta \left(i\right)}\right)}^{l_{\delta \left(i\right)}w_{i}},\prod_{i=1}^{n}{\left(d_{\delta \left(i\right)}\right)}^{l_{\delta \left(i\right)}w_{i}}\right]\rangle \end{array}$$

where $w_{i}$ means the weight coefficient of $\psi_{\delta \left(i\right)}$ such that $0\leq w_{i}\leq 1$ and $\sum_{i=1}^{n}w_{i}=1$ for i=1,2,⋯,n. $\left(\delta \left(1\right),\delta \left(2\right),\cdots ,\delta \left(n\right)\right)$ is a permutation of $\left(1,2,\cdots ,n\right)$, $\psi_{\delta \left(i-1\right)}\geq \psi_{\delta \left(i\right)}$ for i=1,2,⋯,n.

**Definition** **7.** ([49]) *Let* $\psi_{i}=\langle \left[a_{i},b_{i}\right],\left[c_{i},d_{i}\right]\rangle \left(i=1,2,\cdots ,n\right)$ *be n IVPFNs, and* $l_{i}$ *be the self-confidence level of* $\psi_{i}$*. The definition of SC-IVPFWG is as follows:*

(6) $$\begin{array}{l} SC-IVPFWG\left(\left(l_{1},\psi_{1}\right),\left(l_{2},\psi_{2}\right),\cdots ,\left(l_{n},\psi_{n}\right)\right)={⊗}_{i=1}^{n}{\left({\left(\psi_{i}\right)}^{l_{i}}\right)}^{w_{i}}\\ =\langle \left[\prod_{i=1}^{n}{\left(a_{i}\right)}^{l_{i}w_{i}},\prod_{i=1}^{n}{\left(b_{i}\right)}^{l_{i}w_{i}}\right],\left[\sqrt{1-\prod_{i=1}^{n}{\left(1-{\left(c_{i}\right)}^{2}\right)}^{l_{i}w_{i}}},\sqrt{1-\prod_{i=1}^{n}{\left(1-{\left(d_{i}\right)}^{2}\right)}^{l_{i}w_{i}}}\right]\rangle \end{array}$$

where $w_{i}$ is the weight coefficient of $\psi_{i}$ such that $0\leq w_{i}\leq 1$ and $\sum_{i=1}^{n}w_{i}=1$ for i=1,2,⋯,n.

**Definition** **8.** ([49]) *Let* $\psi_{i}=\langle \left[a_{i},b_{i}\right],\left[c_{i},d_{i}\right]\rangle \left(i=1,2,\cdots ,n\right)$ *be n IVPFNs, where* $l_{\delta \left(i\right)}$ *denotes the self-confidence level of* $\psi_{\delta \left(i\right)}$*,* $l_{\delta \left(i\right)}\in \left[0,1\right]$*. The definition of SC-IVPFOWG is as follows:*

(7) $$\begin{array}{c} SC-IVPFOWG\left(\left(l_{1},\psi_{1}\right),\left(l_{2},\psi_{2}\right),\cdots ,\left(l_{n},\psi_{n}\right)\right)={⊗}_{i=1}^{n}{\left({\left(\psi_{\delta (i)}\right)}^{l_{\delta (i)}}\right)}^{w_{i}}\\ =\langle \left[\overset{n}{\underset{i=1}{\prod }}{\left(a_{\delta (i)}\right)}^{l_{\delta (i)}w_{i}},\overset{n}{\underset{i=1}{\prod }}{\left(b_{\delta (i)}\right)}^{l_{\delta (i)}w_{i}}\right],,,\left[\sqrt{1-\overset{n}{\underset{i=1}{\prod }}{\left(1-{\left(c_{\delta (i)}\right)}^{2}\right)}^{l_{\delta (i)}w_{i}}},\sqrt{1-\overset{n}{\underset{i=1}{\prod }}{\left(1-{\left(d_{\delta (i)}\right)}^{2}\right)}^{l_{\delta (i)}w_{i}}}\right]\rangle \end{array}$$

where $w_{i}$ denotes the weight coefficient of $\psi_{\delta \left(i\right)}$ such that $0\leq w_{i}\leq 1$ and $\sum_{i=1}^{n}w_{i}=1$ for i=1,2,⋯,n. $\left(\delta \left(1\right),\delta \left(2\right),\cdots ,\delta \left(n\right)\right)$ is a permutation of $\left(1,2,\cdots ,n\right)$, $\psi_{\delta \left(i-1\right)}\geq \psi_{\delta \left(i\right)}$ for i=1,2,⋯,n.

**Example** **1.** Let the three IVPFNs be $\psi_{1}=\langle \left[0.4472,0.5477\right],\left[0.6325,0.7071\right]\rangle$, $\psi_{2}=\langle \left[0.3162,0.6235\right],\left[0.5477,0.7746\right]\rangle$, and $\psi_{3}=\langle \left[0.3873,0.5916\right],\left[0.5916,0.7416\right]\rangle$, and let their homologous self-confidence level of expert and weight coefficient be denoted by $l={\left(0.7,0.6,0.8\right)}^{T}$ and $w={\left(0.3,0.4,0.3\right)}^{T}$, respectively.

According to Definitions 6 and 8, we obtain the aggregation results as follows:

$$SC-IVPFWA=\langle \left[0.3243,0.5065\right],\left[0.6930,0.8140\right]\rangle$$

$$SC-IVPFWG=\langle \left[0.5102,0.6937\right],\left[0.5064,0.6536\right]\rangle$$

Similarly, we obtain

$$SC-IVPFOWA=\langle \left[0.3369,0.5031\right],\left[0.6959,0.8067\right]\rangle$$

$$SC-IVPFOWG=\langle \left[0.5167,0.6841\right],\left[0.5167,0.6507\right]\rangle$$

According to the above calculation results, there are significant differences in the final aggregation values obtained by the aggregation methods of the SC-IVPFWA (SC-IVPFOWA) and SC-IVPFWG (SC-IVPFOWG) operators. The main reason for this divergence is that SC-IVPFWA (SC-IVPFOWA) is a compromise aggregation method that can effectively neutralize the influence caused by the maximum and minimum values in the aggregation process, whereas the SC-IVPFWG (SC-IVPFOWG) operator mainly reflects individual characteristics and can effectively retain the information of the original data.

The two aggregation methods clearly exhibit their own advantages. To synthesize the advantages of the two methods, next, we present the self-confidence IVPF hybrid weighted average geometric (SC-IVPFHWAG) and self-confidence IVPF ordered hybrid weighted average geometric (SC-IVPFOHWAG) operators.

**Definition** **9.** *Let*$\psi_{i}=\langle \left[a_{i},b_{i}\right],\left[c_{i},d_{i}\right]\rangle \left(i=1,2,\cdots ,n\right)$*be a couple of IVPFNs, where*$l_{i}$*is the self-confidence level of*$\psi_{i}$*. The definition of SC-IVPFHWAG is as follows:*

(8) $$\begin{array}{c} SC-IVPFHWAG\left(\left(l_{1},\psi_{1}\right),\left(l_{2},\psi_{2}\right),\cdots ,\left(l_{n},\psi_{n}\right)\right)={\left({⊕}_{i=1}^{n}w_{i}\left(l_{i}\psi_{i}\right)\right)}^{\theta }{\left({⊗}_{i=1}^{n}{\left({\left(\psi_{i}\right)}^{l_{i}}\right)}^{w_{i}}\right)}^{\left(1-\theta \right)}\\ =\langle \begin{array}{c} \left[{\left(\sqrt{1-\prod_{i=1}^{n}{\left(1-{\left(a_{i}\right)}^{2}\right)}^{l_{i}w_{i}}}\right)}^{\theta }{\left(\prod_{i=1}^{n}{\left(a_{i}\right)}^{l_{i}w_{i}}\right)}^{\left(1-\theta \right)},{\left(\sqrt{1-\prod_{i=1}^{n}{\left(1-{\left(b_{i}\right)}^{2}\right)}^{l_{i}w_{i}}}\right)}^{\theta }{\left(\prod_{i=1}^{n}{\left(b_{i}\right)}^{l_{i}w_{i}}\right)}^{\left(1-\theta \right)}\right],\\ \left[\sqrt{1-{\left(1-\prod_{i=1}^{n}{\left(c_{i}\right)}^{2l_{i}w_{i}}\right)}^{\theta }{\left(\prod_{i=1}^{n}{\left(1-{\left(c_{i}\right)}^{2}\right)}^{l_{i}w_{i}}\right)}^{\left(1-\theta \right)}},\sqrt{1-{\left(1-\prod_{i=1}^{n}{\left(d_{i}\right)}^{2l_{i}w_{i}}\right)}^{\theta }{\left(\prod_{i=1}^{n}{\left(1-{\left(d_{i}\right)}^{2}\right)}^{l_{i}w_{i}}\right)}^{\left(1-\theta \right)}}\right] \end{array}\rangle \end{array}$$

where $w_{i}$ is the weight coefficient of $\psi_{i}$ such that $0\leq w_{i}\leq 1$ and $\sum_{i=1}^{n}w_{i}=1$ for i=1,2,⋯,n, and θ means a real number in the interval $\left[0,1\right]$.

**Proof.**  When n=2, according to Definition 2, we obtain

$$l_{1}\psi_{1}=\langle \left[\sqrt{1-{\left(1-a_{1}^{2}\right)}^{l_{1}}},\sqrt{1-{\left(1-b_{1}^{2}\right)}^{l_{1}}}\right],\left[c_{1}^{l_{1}},d_{1}^{l_{1}}\right]\rangle$$

$$l_{2}\psi_{2}=\langle \left[\sqrt{1-{\left(1-a_{2}^{2}\right)}^{l_{2}}},\sqrt{1-{\left(1-b_{2}^{2}\right)}^{l_{2}}}\right],\left[c_{2}^{l_{2}},d_{2}^{l_{2}}\right]\rangle$$

$$\psi_{1}^{l_{1}}=\langle \left[a_{1}^{l_{1}},b_{1}^{l_{1}}\right],\left[\sqrt{1-{\left(1-c_{1}^{2}\right)}^{l_{1}}},\sqrt{1-{\left(1-d_{1}^{2}\right)}^{l_{1}}}\right]\rangle$$

$$\psi_{2}^{l_{2}}=\langle \left[a_{2}^{l_{2}},b_{2}^{l_{2}}\right],\left[\sqrt{1-{\left(1-c_{2}^{2}\right)}^{l_{2}}},\sqrt{1-{\left(1-d_{2}^{2}\right)}^{l_{2}}}\right]\rangle$$

□

Subsequently,

$$w_{1}\left(l_{1}\psi_{1}\right)=\langle \left[\sqrt{1-{\left(1-a_{1}^{2}\right)}^{l_{1}}{}^{w_{1}}},\sqrt{1-{\left(1-b_{1}^{2}\right)}^{l_{1}}{}^{w_{1}}}\right],\left[c_{1}^{l_{1}w_{1}},d_{1}^{l_{1}w_{1}}\right]\rangle$$

$$w_{2}\left(l_{2}\psi_{2}\right)=\langle \left[\sqrt{1-{\left(1-a_{2}^{2}\right)}^{l_{2}}{}^{w_{2}}},\sqrt{1-{\left(1-b_{2}^{2}\right)}^{l_{2}}{}^{w_{2}}}\right],\left[c_{2}^{l_{2}w_{2}},d_{2}^{l_{2}w_{2}}\right]\rangle$$

$${\left({\left(\psi_{1}\right)}^{l_{1}}\right)}^{w_{1}}=\langle \left[a_{1}^{l_{1}w_{1}},b_{1}^{l_{1}w_{1}}\right],\left[\sqrt{1-{\left(1-c_{1}^{2}\right)}^{l_{1}}{}^{w_{1}}},\sqrt{1-{\left(1-d_{1}^{2}\right)}^{l_{1}}{}^{w_{1}}}\right]\rangle$$

$${\left({\left(\psi_{2}\right)}^{l_{2}}\right)}^{w_{2}}=\langle \left[a_{2}^{l_{2}w_{2}},b_{2}^{l_{2}w_{2}}\right],\left[\sqrt{1-{\left(1-c_{2}^{2}\right)}^{l_{2}}{}^{w_{2}}},\sqrt{1-{\left(1-d_{2}^{2}\right)}^{l_{2}}{}^{w_{2}}}\right]\rangle$$

Thus,

$$\begin{array}{c} SC-IVPFHWAG\left(\left(l_{1},\psi_{1}\right)\left(l_{2},\psi_{2}\right)\right)={\left({⊕}_{i=1}^{2}w_{i}\left(l_{i}\psi_{i}\right)\right)}^{\theta }{\left({⊗}_{i=1}^{2}{\left({\left(\psi_{i}\right)}^{l_{i}}\right)}^{w_{i}}\right)}^{\left(1-\theta \right)}\\ =\left\{\langle \left[\sqrt{1-{{\left(1-a_{1}^{2}\right)}^{l_{1}}}^{w_{1}}},\sqrt{1-{{\left(1-b_{1}^{2}\right)}^{l_{1}}}^{w_{1}}}\right],\left[c_{1}^{l_{1}w_{1}},d_{1}^{l_{1}w_{1}}\right]\rangle ⊕\langle \left[\sqrt{1-{{\left(1-a_{2}^{2}\right)}^{l_{2}}}^{w_{2}}},\sqrt{1-{{\left(1-b_{2}^{2}\right)}^{l_{2}}}^{w_{2}}}\right],\left[c_{2}^{l_{2}w_{2}},d_{2}^{l_{2}w_{2}}\right]\rangle \right\}\\ \times {\left\{\langle \left[a_{1}^{l_{1}w_{1}},b_{1}^{l_{1}w_{1}}\right],\left[\sqrt{1-{\left(1-c_{1}^{2}\right)}^{l_{1}}{}^{w_{1}}},\sqrt{1-{\left(1-d_{1}^{2}\right)}^{l_{1}}{}^{w_{1}}}\right]\rangle ⊗\langle \left[a_{2}^{l_{2}w_{2}},b_{2}^{l_{2}w_{2}}\right],\left[\sqrt{1-{\left(1-c_{2}^{2}\right)}^{l_{2}}{}^{w_{2}}},\sqrt{1-{\left(1-d_{2}^{2}\right)}^{l_{2}}{}^{w_{2}}}\right]\rangle \right\}}^{1-\theta }\\ ={\langle \left[\sqrt{1-{{\left(1-a_{1}^{2}\right)}^{l_{1}}}^{w_{1}}{\left(1-a_{2}^{2}\right)}^{l_{1}w_{1}}},\sqrt{1-{\left(1-b_{1}^{2}\right)}^{l_{2}w_{2}}{\left(1-b_{2}^{2}\right)}^{l_{1}w_{1}}}\right]\left[c_{1}^{l_{1}w_{1}}c_{2}^{l_{2}w_{2}},d_{1}^{l_{1}w_{1}}d_{2}^{l_{2}w_{2}}\right]\rangle }^{\theta }\times \\ {\langle \left[a_{1}^{l_{1}w_{1}}a_{2}^{l_{2}w_{2}},b_{1}^{l_{1}w_{1}}b_{2}^{l_{2}w_{2}}\right]\left[\sqrt{1-{\left(1-c_{1}^{2}\right)}^{l_{1}w_{1}}{\left(1-c_{2}^{2}\right)}^{l_{2}w_{2}}},\sqrt{1-{\left(1-d_{1}^{2}\right)}^{l_{1}w_{1}}{\left(1-d_{2}^{2}\right)}^{l_{2}w_{2}}}\right]\rangle }^{1-\theta }\\ =\langle \begin{array}{c} \left[{\left(\sqrt{1-\prod_{i=1}^{2}{\left(1-{\left(a_{i}\right)}^{2}\right)}^{l_{i}w_{i}}}\right)}^{\theta }{\left(\prod_{i=1}^{2}{\left(a_{i}\right)}^{l_{i}w_{i}}\right)}^{\left(1-\theta \right)},{\left(\sqrt{1-\prod_{i=1}^{2}{\left(1-{\left(b_{i}\right)}^{2}\right)}^{l_{i}w_{i}}}\right)}^{\theta }{\left(\prod_{i=1}^{2}{\left(b_{i}\right)}^{l_{i}w_{i}}\right)}^{\left(1-\theta \right)}\right],\\ \left[\sqrt{1-{\left(1-\prod_{i=1}^{2}{\left(c_{i}\right)}^{2l_{i}w_{i}}\right)}^{\theta }{\left(\prod_{i=1}^{2}{\left(1-{\left(c_{i}\right)}^{2}\right)}^{l_{i}w_{i}}\right)}^{\left(1-\theta \right)}},\sqrt{1-{\left(1-\prod_{i=1}^{2}{\left(d_{i}\right)}^{2l_{i}w_{i}}\right)}^{\theta }{\left(\prod_{i=1}^{2}{\left(1-{\left(d_{i}\right)}^{2}\right)}^{l_{i}w_{i}}\right)}^{\left(1-\theta \right)}}\right] \end{array}\rangle . \end{array}$$

When n=k,

$$\begin{array}{l} SC-IVPFHWAG\left(\left(l_{1},\psi_{1}\right),\left(l_{2},\psi_{2}\right),\cdots ,\left(l_{k},\psi_{k}\right)\right)={\left({⊕}_{i=1}^{k}w_{i}\left(l_{i}\psi_{i}\right)\right)}^{\theta }{\left({⊗}_{i=1}^{k}{\left({\left(\psi_{i}\right)}^{l_{i}}\right)}^{w_{i}}\right)}^{\left(1-\theta \right)}\\ ={\langle \left[\sqrt{1-{{\left(1-a_{1}^{2}\right)}^{l_{1}}}^{w_{1}}{{\left(1-a_{2}^{2}\right)}^{l_{2}}}^{w_{2}}\cdots {{\left(1-a_{k}^{2}\right)}^{l_{k}}}^{w_{k}}},\sqrt{1-{{\left(1-b_{1}^{2}\right)}^{l_{1}}}^{w_{1}}{{\left(1-b_{2}^{2}\right)}^{l_{2}}}^{w_{2}}\cdots {{\left(1-b_{k}^{2}\right)}^{l_{k}}}^{w_{k}}}\right]\left[c_{1}^{l_{1}w_{1}}c_{2}^{l_{2}w_{2}}\cdots c_{k}^{l_{k}w_{k}},d_{1}^{l_{1}w_{1}}d_{2}^{l_{2}w_{2}}\cdots d_{k}^{l_{k}w_{k}}\right]\rangle }^{\theta }\\ \times {\langle \left[a_{1}^{l_{1}w_{1}}a_{2}^{l_{2}w_{2}}\cdots a_{k}^{l_{k}w_{k}},b_{1}^{l_{1}w_{1}}b_{2}^{l_{2}w_{2}}\cdots b_{k}^{l_{k}w_{k}}\right]\left[\sqrt{1-{{\left(1-c_{1}^{2}\right)}^{l_{1}}}^{w_{1}}{{\left(1-c_{2}^{2}\right)}^{l_{2}}}^{w_{2}}\cdots {{\left(1-c_{k}^{2}\right)}^{l_{k}}}^{w_{k}}},\sqrt{1-{{\left(1-d_{1}^{2}\right)}^{l_{1}}}^{w_{1}}{{\left(1-d_{2}^{2}\right)}^{l_{2}}}^{w_{2}}\cdots {{\left(1-d_{k}^{2}\right)}^{l_{k}}}^{w_{k}}}\right]\rangle }^{1-\theta }\\ =\langle \begin{array}{c} \left[{\left(\sqrt{1-\prod_{i=1}^{k}{\left(1-{\left(a_{i}\right)}^{2}\right)}^{l_{i}w_{i}}}\right)}^{\theta }{\left(\prod_{i=1}^{k}{\left(a_{i}\right)}^{l_{i}w_{i}}\right)}^{\left(1-\theta \right)},{\left(\sqrt{1-\prod_{i=1}^{k}{\left(1-{\left(b_{i}\right)}^{2}\right)}^{l_{i}w_{i}}}\right)}^{\theta }{\left(\prod_{i=1}^{k}{\left(b_{i}\right)}^{l_{i}w_{i}}\right)}^{\left(1-\theta \right)}\right],\\ \left[\sqrt{1-{\left(1-\prod_{i=1}^{k}{\left(c_{i}\right)}^{2l_{i}w_{i}}\right)}^{\theta }{\left(\prod_{i=1}^{k}{\left(1-{\left(c_{i}\right)}^{2}\right)}^{l_{i}w_{i}}\right)}^{\left(1-\theta \right)}},\sqrt{1-{\left(1-\prod_{i=1}^{k}{\left(d_{i}\right)}^{2l_{i}w_{i}}\right)}^{\theta }{\left(\prod_{i=1}^{k}{\left(1-{\left(d_{i}\right)}^{2}\right)}^{l_{i}w_{i}}\right)}^{\left(1-\theta \right)}}\right] \end{array}\rangle . \end{array}$$

Furthermore, when n=k+1, we obtain

$$\begin{array}{l} SC-IVPFHWAG\left(\left(l_{1},\psi_{1}\right),\left(l_{2},\psi_{2}\right),\cdots ,\left(l_{k},\psi_{k}\right)\left(l_{k+1},\psi_{k+1}\right)\right)={\left({⊕}_{i=1}^{k+1}w_{i}\left(l_{i}\psi_{i}\right)\right)}^{\theta }{\left({⊗}_{i=1}^{k+1}{\left({\left(\psi_{i}\right)}^{l_{i}}\right)}^{w_{i}}\right)}^{\left(1-\theta \right)}\\ ={\langle \left[\begin{array}{l} \sqrt{1-{\left(1-a_{1}^{2}\right)}^{l_{1}}{}^{w_{1}}{\left(1-a_{2}^{2}\right)}^{l_{2}}{}^{w_{2}}\cdots {\left(1-a_{k}^{2}\right)}^{l_{k}}{}^{w_{k}}{\left(1-a_{k+1}^{2}\right)}^{l_{k+1}}{}^{w_{k+1}}},\\ \sqrt{1-{\left(1-b_{1}^{2}\right)}^{l_{1}}{}^{w_{1}}{\left(1-b_{2}^{2}\right)}^{l_{2}}{}^{w_{2}}\cdots {\left(1-b_{k}^{2}\right)}^{l_{k}}{}^{w_{k}}{\left(1-b_{k+1}^{2}\right)}^{l_{k+1}}{}^{w_{k+1}}} \end{array}\right],\left[c_{1}^{l_{1}w_{1}}c_{2}^{l_{2}w_{2}}\cdots c_{k}^{l_{k}w_{k}}c_{k+1}^{l_{k+1}w_{k+1}},d_{1}^{l_{1}w_{1}}d_{2}^{l_{2}w_{2}}\cdots d_{k}^{l_{k}w_{k}}d_{k+1}^{l_{k+1}w_{k+1}}\right]\rangle }^{\theta }\\ \times \langle \left[a_{1}^{l_{1}w_{1}}a_{2}^{l_{2}w_{2}}\cdots a_{k}^{l_{k}w_{k}}a_{k+1}^{l_{k+1}w_{k+1}},b_{1}^{l_{1}w_{1}}b_{2}^{l_{2}w_{2}}\cdots b_{k}^{l_{k}w_{k}}b_{k+1}^{l_{k+1}w_{k+1}}\right]\left[\begin{array}{l} \sqrt{1-{\left(1-c_{1}^{2}\right)}^{l_{1}}{}^{w_{1}}{\left(1-c_{2}^{2}\right)}^{l_{2}}{}^{w_{2}}\cdots {\left(1-c_{k}^{2}\right)}^{l_{k}}{}^{w_{k}}{\left(1-c_{k+1}^{2}\right)}^{l_{k+1}}{}^{w_{k+1}}},\\ \sqrt{1-{\left(1-d_{1}^{2}\right)}^{l_{1}}{}^{w_{1}}{\left(1-d_{2}^{2}\right)}^{l_{2}}{}^{w_{2}}\cdots {\left(1-d_{k}^{2}\right)}^{l_{k}}{}^{w_{k}}{\left(1-d_{k+1}^{2}\right)}^{l_{k+1}}{}^{w_{k+1}}} \end{array}\right]\rangle \\ =\langle \begin{array}{c} \left[{\left(\sqrt{1-\prod_{i=1}^{k+1}{\left(1-{\left(a_{i}\right)}^{2}\right)}^{l_{i}w_{i}}}\right)}^{\theta }{\left(\prod_{i=1}^{k+1}{\left(a_{i}\right)}^{l_{i}w_{i}}\right)}^{\left(1-\theta \right)},{\left(\sqrt{1-\prod_{i=1}^{k+1}{\left(1-{\left(b_{i}\right)}^{2}\right)}^{l_{i}w_{i}}}\right)}^{\theta }{\left(\prod_{i=1}^{k+1}{\left(b_{i}\right)}^{l_{i}w_{i}}\right)}^{\left(1-\theta \right)}\right],\\ \left[\sqrt{1-{\left(1-\prod_{i=1}^{k+1}{\left(c_{i}\right)}^{2l_{i}w_{i}}\right)}^{\theta }{\left(\prod_{i=1}^{k+1}{\left(1-{\left(c_{i}\right)}^{2}\right)}^{l_{i}w_{i}}\right)}^{\left(1-\theta \right)}},\sqrt{1-{\left(1-\prod_{i=1}^{k+1}{\left(d_{i}\right)}^{2l_{i}w_{i}}\right)}^{\theta }{\left(\prod_{i=1}^{k+1}{\left(1-{\left(d_{i}\right)}^{2}\right)}^{l_{i}w_{i}}\right)}^{\left(1-\theta \right)}}\right] \end{array}\rangle . \end{array}$$

That is, Equation (8) holds true, regardless of the value of *n*.

**Remark** **1.** *When*θ=0*, SC-IVPFHWAG degenerates into the SC-IVPFWG operator* [49]*:*

$$\begin{array}{c} SC-IVPFHWAG\left(\left(l_{1},\psi_{1}\right),\left(l_{2},\psi_{2}\right),\cdots ,\left(l_{n},\psi_{n}\right)\right)={\left({⊕}_{i=1}^{n}w_{i}\left(l_{i}\psi_{i}\right)\right)}^{0}{\left({⊗}_{i=1}^{n}{\left({\left(\psi_{i}\right)}^{l_{i}}\right)}^{w_{i}}\right)}^{\left(1-0\right)}\\ =\langle \left[\left(\prod_{i=1}^{n}{\left(a_{i}\right)}^{l_{i}w_{i}}\right),\left(\prod_{i=1}^{n}{\left(b_{i}\right)}^{l_{i}w_{i}}\right)\right],\left[\sqrt{1-{\left(\prod_{i=1}^{n}{\left(1-{\left(c_{i}\right)}^{2}\right)}^{l_{i}w_{i}}\right)}^{1}},\sqrt{1-{\left(\prod_{i=1}^{n}{\left(1-{\left(d_{i}\right)}^{2}\right)}^{l_{i}w_{i}}\right)}^{1}}\right]\rangle \\ =SC-IVPFWG. \end{array}$$

**Remark** **2.** *When*θ=1*, SC-IVPFHWAG degenerates into the SC-IVPFWA operator* [49]*, that is,*

$$\begin{array}{c} SC-IVPFHWAG\left(\left(l_{1},\psi_{1}\right),\left(l_{2},\psi_{2}\right),\cdots ,\left(l_{n},\psi_{n}\right)\right)={\left({⊕}_{i=1}^{n}w_{i}\left(l_{i}\psi_{i}\right)\right)}^{1}{\left({⊗}_{i=1}^{n}{\left({\left(\psi_{i}\right)}^{l_{i}}\right)}^{w_{i}}\right)}^{\left(1-1\right)}\\ =\langle \left[\left(\sqrt{1-\prod_{i=1}^{n}{\left(1-{\left(a_{i}\right)}^{2}\right)}^{l_{i}w_{i}}}\right),\left(\sqrt{1-\prod_{i=1}^{n}{\left(1-{\left(b_{i}\right)}^{2}\right)}^{l_{i}w_{i}}}\right)\right],\left[\sqrt{1-\left(1-\prod_{i=1}^{n}{\left(c_{i}\right)}^{2l_{i}w_{i}}\right)},\sqrt{1-\left(1-\prod_{i=1}^{n}{\left(d_{i}\right)}^{2l_{i}w_{i}}\right)}\right]\rangle \\ =SC-IVPFWA. \end{array}$$

Therefore, we have proven that the proposed SC-IVPFHWAG operator is a general form of the existing SC-IVPFWA and SC-IVPFWG operators.

**Definition** **10.** *Let*$\psi_{i}=\langle \left[a_{i},b_{i}\right],\left[c_{i},d_{i}\right]\rangle \left(i=1,2,\cdots ,n\right)$*be a couple of IVPFNs, where*$l_{\delta \left(i\right)}$*denotes the self-confidence level of*$\psi_{\delta \left(i\right)}$*,*$l_{\delta \left(i\right)}\in \left[0,1\right]$. $\left(\delta \left(1\right),\delta \left(2\right),\cdots ,\delta \left(n\right)\right)$*is a permutation of*$\left(1,2,\cdots ,n\right)$*,*$\psi_{\delta \left(i-1\right)}\geq \psi_{\delta \left(i\right)}$*for*i=1,2,⋯,n*. The definition of SC-IVPFOHWAG is as follows:*

(9) $$\begin{array}{c} SC-IVPFOHWAG\left(\left(l_{1},\psi_{1}\right),\left(l_{2},\psi_{2}\right),\cdots ,\left(l_{n},\psi_{n}\right)\right)={\left({⊕}_{i=1}^{n}w_{i}\left(l_{\delta \left(i\right)}\psi_{\delta \left(i\right)}\right)\right)}^{\theta }{\left({⊗}_{i=1}^{n}{\left({\left(\psi_{\delta \left(i\right)}\right)}^{l_{\delta \left(i\right)}}\right)}^{w_{i}}\right)}^{\left(1-\theta \right)}\\ =\langle \begin{array}{c} \left[{\left(\sqrt{1-\prod_{i=1}^{n}{\left(1-{\left(a_{\delta \left(i\right)}\right)}^{2}\right)}^{l_{\delta \left(i\right)}w_{i}}}\right)}^{\theta }{\left(\prod_{i=1}^{n}{\left(a_{\delta \left(i\right)}\right)}^{l_{\delta \left(i\right)}w_{i}}\right)}^{\left(1-\theta \right)},{\left(\sqrt{1-\prod_{i=1}^{n}{\left(1-{\left(b_{\delta \left(i\right)}\right)}^{2}\right)}^{l_{\delta \left(i\right)}w_{i}}}\right)}^{\theta }{\left(\prod_{i=1}^{n}{\left(b_{\delta \left(i\right)}\right)}^{l_{\delta \left(i\right)}w_{i}}\right)}^{\left(1-\theta \right)}\right],\\ \left[\sqrt{1-{\left(1-\prod_{i=1}^{n}{\left(c_{\delta \left(i\right)}\right)}^{2l_{\delta \left(i\right)}w_{i}}\right)}^{\theta }{\left(\prod_{i=1}^{n}{\left(1-{\left(c_{\delta \left(i\right)}\right)}^{2}\right)}^{l_{\delta \left(i\right)}w_{i}}\right)}^{\left(1-\theta \right)}},\sqrt{1-{\left(1-\prod_{i=1}^{n}{\left(d_{\delta \left(i\right)}\right)}^{2l_{\delta \left(i\right)}w_{i}}\right)}^{\theta }{\left(\prod_{i=1}^{n}{\left(1-{\left(d_{\delta \left(i\right)}\right)}^{2}\right)}^{l_{\delta \left(i\right)}w_{i}}\right)}^{\left(1-\theta \right)}}\right] \end{array}\rangle \end{array}$$

where $w_{i}$ denotes the weight coefficient of $\psi_{\delta \left(i\right)}$ such that $0\leq w_{i}\leq 1$ and $\sum_{i=1}^{n}w_{i}=1$ for i=1,2,⋯,n.

**Remark** **3.** *When*θ=0*, the SC-IVPFOHWAG is deduced into the SC-IVPFOWG* [49]*, which can be proven by the following:*

$$\begin{array}{c} SC-IVPFOHWAG\left(\left(l_{1},\psi_{1}\right),\left(l_{2},\psi_{2}\right),\cdots ,\left(l_{n},\psi_{n}\right)\right)={\left({⊕}_{i=1}^{n}w_{i}\left(l_{\delta \left(i\right)}\psi_{\delta \left(i\right)}\right)\right)}^{0}{\left({⊗}_{i=1}^{n}{\left({\left(\psi_{\delta \left(i\right)}\right)}^{l_{\delta \left(i\right)}}\right)}^{w_{i}}\right)}^{\left(1-0\right)}\\ =\langle \begin{array}{c} \left[{\left(\sqrt{1-\prod_{i=1}^{n}{\left(1-{\left(a_{\delta \left(i\right)}\right)}^{2}\right)}^{l_{\delta \left(i\right)}w_{i}}}\right)}^{0}{\left(\prod_{i=1}^{n}{\left(a_{\delta \left(i\right)}\right)}^{l_{\delta \left(i\right)}w_{i}}\right)}^{1},{\left(\sqrt{1-\prod_{i=1}^{n}{\left(1-{\left(b_{\delta \left(i\right)}\right)}^{2}\right)}^{l_{\delta \left(i\right)}w_{i}}}\right)}^{0}{\left(\prod_{i=1}^{n}{\left(b_{\delta \left(i\right)}\right)}^{l_{\delta \left(i\right)}w_{i}}\right)}^{1}\right],\\ \left[\sqrt{1-{\left(1-\prod_{i=1}^{n}{\left(c_{\delta \left(i\right)}\right)}^{2l_{\delta \left(i\right)}w_{i}}\right)}^{0}{\left(\prod_{i=1}^{n}{\left(1-{\left(c_{\delta \left(i\right)}\right)}^{2}\right)}^{l_{\delta \left(i\right)}w_{i}}\right)}^{1}},\sqrt{1-{\left(1-\prod_{i=1}^{n}{\left(d_{\delta \left(i\right)}\right)}^{2l_{\delta \left(i\right)}w_{i}}\right)}^{0}{\left(\prod_{i=1}^{n}{\left(1-{\left(d_{\delta \left(i\right)}\right)}^{2}\right)}^{l_{\delta \left(i\right)}w_{i}}\right)}^{1}}\right] \end{array}\rangle \\ =\langle \left[\left(\prod_{i=1}^{n}{\left(a_{\delta \left(i\right)}\right)}^{l_{\delta \left(i\right)}w_{i}}\right),\left(\prod_{i=1}^{n}{\left(b_{\delta \left(i\right)}\right)}^{l_{\delta \left(i\right)}w_{i}}\right)\right],\left[\sqrt{1-\left(\prod_{i=1}^{n}{\left(1-{\left(c_{\delta \left(i\right)}\right)}^{2}\right)}^{l_{\delta \left(i\right)}w_{i}}\right)},\sqrt{1-\left(\prod_{i=1}^{n}{\left(1-{\left(d_{\delta \left(i\right)}\right)}^{2}\right)}^{l_{\delta \left(i\right)}w_{i}}\right)}\right]\rangle \\ =SC-IVPFOWG. \end{array}$$

**Remark** **4.** *When*θ=1*, SC-IVPFOHWAG is deduced into the SC-IVPFOWA operator* [49]*:*

$$\begin{array}{c} SC-IVPFOHWAG\left(\left(l_{1},\psi_{1}\right),\left(l_{2},\psi_{2}\right),\cdots ,\left(l_{n},\psi_{n}\right)\right)={\left({⊕}_{i=1}^{n}w_{i}\left(l_{\delta \left(i\right)}\psi_{\delta \left(i\right)}\right)\right)}^{1}{\left({⊗}_{i=1}^{n}{\left({\left(\psi_{\delta \left(i\right)}\right)}^{l_{\delta \left(i\right)}}\right)}^{w_{i}}\right)}^{\left(1-1\right)}\\ =\langle \begin{array}{c} \left[{\left(\sqrt{1-\prod_{i=1}^{n}{\left(1-{\left(a_{\delta \left(i\right)}\right)}^{2}\right)}^{l_{\delta \left(i\right)}w_{i}}}\right)}^{1}{\left(\prod_{i=1}^{n}{\left(a_{\delta \left(i\right)}\right)}^{l_{\delta \left(i\right)}w_{i}}\right)}^{0},{\left(\sqrt{1-\prod_{i=1}^{n}{\left(1-{\left(b_{\delta \left(i\right)}\right)}^{2}\right)}^{l_{\delta \left(i\right)}w_{i}}}\right)}^{1}{\left(\prod_{i=1}^{n}{\left(b_{\delta \left(i\right)}\right)}^{l_{\delta \left(i\right)}w_{i}}\right)}^{0}\right],\\ \left[\sqrt{1-{\left(1-\prod_{i=1}^{n}{\left(c_{\delta \left(i\right)}\right)}^{2l_{\delta \left(i\right)}w_{i}}\right)}^{1}{\left(\prod_{i=1}^{n}{\left(1-{\left(c_{\delta \left(i\right)}\right)}^{2}\right)}^{l_{\delta \left(i\right)}w_{i}}\right)}^{0}},\sqrt{1-{\left(1-\prod_{i=1}^{n}{\left(d_{\delta \left(i\right)}\right)}^{2l_{\delta \left(i\right)}w_{i}}\right)}^{1}{\left(\prod_{i=1}^{n}{\left(1-{\left(d_{\delta \left(i\right)}\right)}^{2}\right)}^{l_{\delta \left(i\right)}w_{i}}\right)}^{0}}\right] \end{array}\rangle \\ =\langle \left[\left(\sqrt{1-\prod_{i=1}^{n}{\left(1-{\left(a_{\delta \left(i\right)}\right)}^{2}\right)}^{l_{\delta \left(i\right)}w_{i}}}\right),\left(\sqrt{1-\prod_{i=1}^{n}{\left(1-{\left(b_{\delta \left(i\right)}\right)}^{2}\right)}^{l_{\delta \left(i\right)}w_{i}}}\right)\right],\left[\sqrt{1-\left(1-\prod_{i=1}^{n}{\left(c_{\delta \left(i\right)}\right)}^{2l_{\delta \left(i\right)}w_{i}}\right)},\sqrt{1-\left(1-\prod_{i=1}^{n}{\left(d_{\delta \left(i\right)}\right)}^{2l_{\delta \left(i\right)}w_{i}}\right)}\right]\rangle \\ =SC-IVPFOWA. \end{array}$$

Thus, we have proven that the existing SC-IVPFOWA and SC-IVPFOWG operators are particular cases of the proposed SC-IVPFOHWAG operator.

**Example** **2.** (Continuing with Example 1). There are three IVPFNs: $\psi_{1}=\langle \left[0.4472,0.5477\right],\left[0.6325,0.7071\right]\rangle$, $\psi_{2}=\langle \left[0.3162,0.6235\right],\left[0.5477,0.7746\right]\rangle$, and $\psi_{3}=\langle \left[0.3873,0.5916\right],\left[0.5916,0.7416\right]\rangle$, and their corresponding self-confidence levels of experts and weight coefficients are denoted by $l={\left(0.7,0.6,0.8\right)}^{T}$ and $w={\left(0.3,0.4,0.3\right)}^{T}$, respectively. Let θ=0.5. According to Definition 9, we obtain $SC-IVPFHWAG\left(\left(l_{1},\psi_{1}\right),\left(l_{2},\psi_{2}\right),\left(l_{3},\psi_{3}\right)\right)=\langle \left[0.4068,0.5927\right],\left[0.6151,0.7485\right]\rangle$.

Referring to Definition 10, we can calculate $SC-IVPFOHWAG\left(\left(l_{1},\psi_{1}\right),\left(l_{2},\psi_{2}\right),\left(l_{3},\psi_{3}\right)\right)=\langle \left[0.4172,0.5867\right],\left[0.6206,0.7425\right]\rangle .$ It is obvious that there is a significant difference in the aggregation results obtained by the SC-IVPFHWAG (SC-IVPFOHWAG), SC-IVPFWA (SC-IVPFOWA), and SC-IVPFWG (SC-IVPFOWG) operators in Example 1. The main reason is that the proposed SC-IVPFHWAG (SC-IVPFOHWAG) operator can effectively integrate the weighted and geometric average methods, and the final aggregation results are highly coincident. Obviously, the SC-IVPFHWAG and SC-IVPFOHWAG operators generalize the existing self-confidence IVPF aggregation operators, thereby retaining their superiority. Moreover, the SC-IVPFHWAG (SC-IVPFOHWAG) operator can provide a flexible form by controlling the values of the parameter θ in the decision-making process, which provides more options for decision-makers to select an appropriate aggregation method according to their own judgment needs.

## 5. Decision Making for Green Recycling Supplier Selection of Shared Bicycles

### 5.1. Evaluation Index System for the Selection of Shared Bicycle Green Recycling Suppliers

There are a large number of studies about the assessment criteria for green supplier selection. Generally, the determination of assessment criteria mainly focuses on three dimensions such as economy, environment, and society. The economic dimension intends to maximize revenue inflow and minimize resource outflow [50]. The environmental dimension intends to alleviate resource consumption and pollution. Lastly, the social dimension mainly includes the government and the public. Referring to the relevant literature [1,2,5,20,51,52,53,54,55,56,57,58,59,60,61,62,63] and combining the operation process of shared bicycles [1], this paper innovatively constructs a novel evaluation index system for the selection of shared bicycle green recycling suppliers. The details are shown in Table 1.

Financial ability ($H_{1}$): The development of recycling activities of shared bicycles is often accompanied by multiple links such as search, transportation, decomposition, and processing. Each link requires a lot of manpower and material resources, and the operation cycle is long, which requires a solid financial reserve. Once a certain link is suspended due to insufficient funds, the overall recycling process will be interrupted, and it will be difficult to match the increasing number of recycled vehicles, which will lead to the failure of the entire operation of shared bicycles. For recycling service providers, a sound and stable financial position can greatly enhance the overall competitive advantage. Therefore, the financial ability of the supplier is also one of the factors that we need to focus on when selecting a supplier.

Cost control ability ($H_{2}$): The operating mechanism of an enterprise is reflected in the cost control ability. Under the premise of ensuring the normal and reasonable operation of the enterprise, both the minimum consumption of internal resources and the optimal cost control are realized.

Quality assurance ($H_{3}$) shows the external manifestation of the enterprise’s recycling service capability, that is, the failure rate and rejection rate of recycled products. Once the failure rate and the rejection rate are very high, it means that the recycling technology of the enterprise does not meet the standard, and it is difficult to realize the rational use of resources and meet the economic requirements.

Green image ($H_{4}$) focuses on the following expression: The increasing spread of the concept of green travel has made shared bicycles widely utilized, but while people’s daily travel is becoming more and more convenient, it is also accompanied by the failure and abandonment of a large number of shared bicycles. This situation not only causes a waste of resources but also has an irreversible impact on the overall social environment. Therefore, in the context of advocating green China, recycling services for shared bicycles are needed while building a low-carbon city. On this basis, shared bicycle recycling service providers are supposed to possess an intensive awareness of environmental protection and green business philosophy. The green image shall be determined as the standard for selecting green recycling suppliers of shared bicycles.

Pollution and emission ($H_{5}$): The purpose of selecting third-party green suppliers in this paper is to reduce the negative impact on the environment. If the operation of recycling enterprises itself will cause serious pollution to the environment, the mitigation of waste bicycle recycling to the environment will also be greatly reduced. Therefore, it is required that the pollution and exhaust emissions generated by the operation of third-party recycling suppliers should be within the range reasonably permitted by the state, and the weaker the impact on the environment, the better the green supplier is.

Recovery capacity ($H_{6}$): This article focuses on recycling services from third-party suppliers. As the name implies, recovery capacity is a key factor in evaluating whether a supplier is up to this task. Recovery capacity mainly refers to the supplier’s recycling quantity per hour, the overall recycling rate, and the unit recycling cost. This indicator can not only reflect the overall operation scale of the supplier enterprise but also reflect the execution efficiency and planning ability of the enterprise. Therefore, it is regarded as an indispensable attribute to determine a suitable supplier.

Search ability ($H_{7}$): The premise of measuring the overall level of a supplier’s recycling service is to pay attention to its ability to identify and search for faulty vehicles. Due to the convenience of shared bicycles, their parking places are often not in a fixed parking area but scattered in the corners of the city without regularity. This requires suppliers to possess certain technical means to accurately judge their specific parking area, which can not only greatly save the overall recovery cost but also shorten the search time to a great extent. Therefore, a compliant supplier should be with mature search ability.

Cooperation with government ($H_{8}$): The standardized operation of an enterprise is inseparable from the support of the policy, and the recycling business of the enterprise should comply with the relevant regulations of the local government and be carried out within a reasonable scope. The enterprises should be very familiar with the relevant policies, laws, regulations, and specific regulations issued by the government to ensure that the operation of enterprises does not run counter to local policies.

Publicity and education ($H_{9}$): Enterprises should have a certain knowledge of publicity and education. Not only hold the awareness of environmental protection in the actual operation process but also guide the awareness of environmental protection in the process of enterprise publicity and customer participation.

### 5.2. MADM Framework Based on SC-IVPFHWAG and SC-IVPFOHWAG Operators

In this section, we discuss the application of the SC-IVPFHWAG and SC-IVPFOHWAG operators in a MADM problem. Suppose that the alternative set is $A=\left\{A_{1},A_{2},\cdots ,A_{m}\right\}$ and the relevant attribute set is $C=\left\{C_{1},C_{2},\cdots ,C_{n}\right\}$. Experts provided their opinions or assessment under *n* attributes for each alternative. To reflect the vagueness and imprecision in the evaluation process accurately, the IVPFNs $\psi_{ij}=\langle \left[a_{ij},b_{ij}\right],\left[c_{ij},d_{ij}\right]\rangle \left(i=1,2,\cdots ,m;j=1,2,\cdots ,n\right)$ are used to express the evaluation information, where $\left[a_{ij},b_{ij}\right]$ represents the membership interval-valued information for alternative $A_{i}$ under attribute $C_{j}$, and $\left[c_{ij},d_{ij}\right]$ denotes the non-membership interval-valued information for alternative $A_{i}$ under attribute $C_{j}$. Based on the experts’ familiarity with the attributes, the corresponding self-confidence levels are expressed by $l={\left(l_{1},l_{2},\cdots ,l_{n}\right)}^{T}$. The weight coefficient of the attributes is $w={\left(w_{1},w_{2},\cdots ,w_{n}\right)}^{T}$, satisfying $\sum_{i=1}^{n}w_{i}=1$. The main steps of the proposed MADM method are as follows:

**Step 1.** Construct the IVPF decision matrix $P={\left(\psi_{ij}\right)}_{m\times n}(i=1,2,\cdots ,m;j=1,2,\cdots ,n)$.

**Step 2.** Normalize the IVPF decision matrix. When $C_{j}$ is a benefit attribute, $\tilde{\psi_{ij}}=\psi_{ij}$; when $C_{j}$ is a cost attribute, $\tilde{\psi_{ij}}={\left(\psi_{ij}\right)}^{c}$.

**Step 3.** Apply the SC-IVPFHWAG (or SC-IVPFOHWAG) operator to obtain the aggregated values ${ϒ}_{i}\left(i=1,2,\cdots ,m\right)$ for each alternative $A_{i}$.

**Step 4.** Sort the alternatives according to ${ϒ}_{i}\left(i=1,2,\cdots ,m\right)$ and choose one with best performance.

## 6. An Illustrative Example

The content of this partition is mainly composed of three aspects, which are the practical case of the selection of shared bicycle recycling suppliers, the sensitivity analysis of the parameter, and the comparative study with other methods. Through the detailed analysis and research, the feasibility and practicability of the method proposed in this paper are further verified.

### 6.1. Practical Case

With the improvement in the overall economic level of society, the number of motor vehicles in cities is increasing. A large number of motor vehicles facilitates people’s daily travel but also causes problems such as an increase in the urban congestion rate, frequent traffic accidents, and degraded air quality. This results in negative effects on the safety of life and property. The 19th National Congress of the Communist Party of China advocated protecting the ecological environment, building a beautiful China, and promoting steady economic development. As a typical example of low-carbon travel, bicycles play a significant role in urban transportation systems. Moreover, information technology is gradually being applied to all areas of society, and the sharing economy has emerged over time. Under the sharing economy model, shared bicycle services have attracted widespread attention, as they can effectively alleviate traffic and road congestion towards building a clean, low-carbon, safe, and efficient traveling system. However, when the number of faulty shared bicycles is too large, maintenance will not be timely, and vehicles will be abandoned, resulting in a waste of resources and environmental pollution. Excessive abandoned vehicles will result in the negative impact outweighing the positive one of shared bicycles. It is therefore imperative to implement recycling services for shared bicycles. It is not only an enthusiastic response to the construction of low-carbon cities but a strategical implementation for shared bicycle operators under the leadership of sustainable development. Moreover, the selection of third-party suppliers exerts a significant role in the service experience of the user and the operational performance of the enterprise.

**Figure 1:**
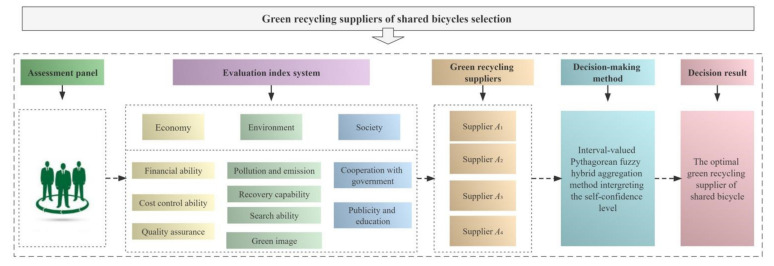
An illustrative diagram for green recycling supplier selection of shared bicycles.

The proposed decision method was applied to the green recycling supplier selection of shared bicycles to improve operational efficiency and strengthen environmental governance. Referring to the proposed evaluation index system in Section 5.1, this paper utilizes nine criteria under three dimensions to assess and select shared bicycle recycling suppliers, namely, financial ability ($H_{1}$), cost control ability ($H_{2}$), quality assurance ($H_{3}$), green image ($H_{4}$), pollution and emission ($H_{5}$), recovery capability ($H_{6}$), search ability ($H_{7}$), cooperation with government ($H_{8}$), and publicity and education ($H_{9}$). An illustrative diagram for green recycling supplier selection of shared bicycles is exhibited in Figure 1.

The proposed evaluation model is confirmed in depth through a contradistinctive analysis of four green suppliers: $A_{1}$, $A_{2}$, $A_{3}$, and $A_{4}$. Among them, $A_{1}$ is an enterprise engaged in the recovery and reuse of various renewable resources, adhering to the business philosophy of “sincere cooperation, credit management, high price peer, committed to the cause of environmental protection”. Its main recovery projects include: Locator chip lock, recycling shared bikes, recycling shared bicycle lithium batteries, recycling all kinds of shared bike locks, and so on. $A_{2}$ is a green environmental protection enterprise specializing in the comprehensive disposal of hazardous waste. In terms of the disposal category, it can undertake 41 major categories and 431 small categories of safe disposal in the new 2021 Hazard list. At the same time, it also undertakes a package of “nanny-style butler services” such as external consultation, technical and on-site engineering operations, and socialized emergency rescue services through environmental service projects and innovative environmental protection projects. $A_{3}$ is a solid waste disposal company, mainly engaged in the reception, disposal, landfill, comprehensive utilization, and technical consulting services of general solid waste (excluding hazardous waste); environmental restoration and treatment; promotion and service of energy-saving and environmental protection technology. $A_{4}$ is a collection of research and development, investment, operation, design, construction, consulting, and equipment manufacturing in one with a sound industrial chain, is a “full value chain” and “one-stop” environmental service supplier in the field of solid waste treatment. Its national high-tech enterprises specializing in solid waste disposal are committed to providing a safe, efficient, and economical overall solution for waste disposal.

The assessment panel is composed of well-known experts in the industry, including doctors of Engineering in the field of resource recovery, the expert member of a resource association, the expert member of a renewable resources industry alliance, the member of an environmental research institute, the expert of sharing economy, the general manager of a bicycle enterprise. The specific assessment information for each criterion from the assessment panel is presented in Table 2. To draw a comprehensive and accurate conclusion, the corresponding self-confidence level was obtained based on the familiarity of the assessment panel with the criteria; that is, $l={\left(0.6,0.4,0.7,0.5,0.6,0.7,0.8,0.4,0.7\right)}^{T}$, and the weight coefficient is $w={\left(0.15,0.09,0.13,0.12,0.07,0.08,0.06,0.14,0.16\right)}^{T}$.

**Step 1.** The evaluation attributes are beneficial; therefore, there is no need to normalize the IVPF decision matrix.

**Step 2.** The aggregation results of each alternative are obtained using the proposed SC-IVPFHWAG and SC-IVPFOHWAG operators, as indicated in Table 3.

**Step 3.** The four suppliers are ranked according to the aggregation results, and the specific results are presented in Table 4.

**Step 4.** $A_{2}$ is selected as the most desirable supplier to carry out recycling services.

### 6.2. Sensitivity Analysis

It is supposed that parameter θ=0.5 in the above example of green recycling supplier selection for shared bicycles. However, the value of parameter θ is variable determined by the decision-maker according to the practical demands. Obviously, the final ranking order would be transformed if the parameter θ is changed. To further discuss the influence of parameter θ on the selection of optimal green supplier of shared bicycles, in the following, we explore the variation in the ranking order of the four suppliers by the SC-IVPFHWAG and SC-IVPFOHWAG operators, and the specific information is given in Table 5 and Table 6, respectively.

From Table 5, it is evident that when the value of the parameter θ changes constantly, the supplier ranking outcomes obtained by SC-IVPFHWAG operator are variable. The best one is identical as $A_{2}$ in four suppliers no matter what the parameter θ is. More narrowly, the corresponding ranking order is $A_{2}≻A_{3}≻A_{1}≻A_{4}$ while 0≤θ≤0.05, $A_{4}$ is the least likely option among the four suppliers. For 0.1≤θ≤1, the relevant ranking orders are $A_{2}≻A_{3}≻A_{4}≻A_{1}$, showing the judgement of the best supplier and the worst one are identical, i.e., $A_{2}$ and $A_{1}$. When the parameter θ is 0, the SC-IVPFHWAG operator degenerates into SC-IVPFWG operator, and when the parameter is 1, the SC-IVPFHWAG operator degenerates into SC-IVPFWA operator.

**Figure 2:**
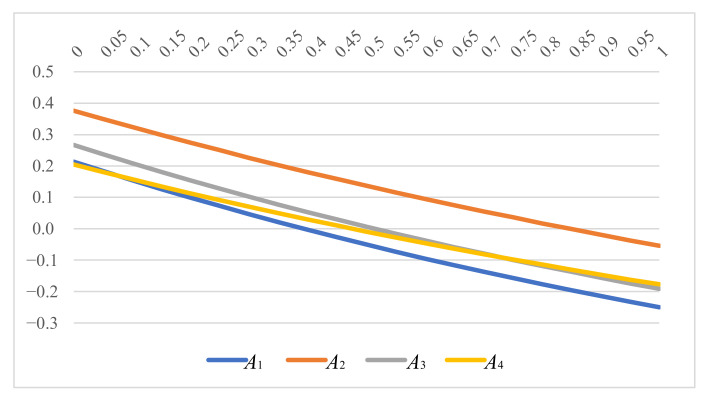
Variation by parameter θ.

In Figure 2, the dynamic changing of the alternative rankings obtained by SC-IVPFHWAG operator due to different parameter values are shown. We preserve that the score values of the four alternatives decrease when the value of parameter θ increases, the overall dynamic changes are presented in an downward trend, but the score value decreasing speed of $A_{4}$ is less than $A_{1}$ and $A_{3}$. Consequently, Figure 2 shows that the comprehensive ranking of $A_{4}$ increases gradually with the continuous increase in the value of parameter θ. In detail, when the parameter θ changes to 0.1, the score value of $A_{4}$ is higher than that of $A_{1}$, and when parameter θ increases to 0.75, the score value of $A_{4}$ is higher than that of $A_{3}$, and $A_{4}$ changes into the second ranking order while $A_{3}$ degenerates into the third ranking order. At this point, the arrangement of four academies tends to stable.

**Figure 3:**
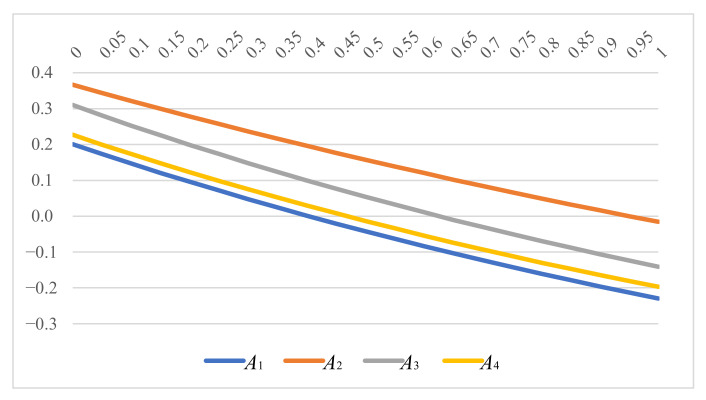
Variation by parameter θ.

Table 6 gives the ranking results of four suppliers acquired by SC-IVPFOHWAG operator of the parameter θ. It is shown that the ranking of the four suppliers remains stable no matter how the value of parameter θ changes, and the final ranking outcome is $A_{2}≻A_{3}≻A_{4}≻A_{1}$. Similarly, we plot the intuitive changing comparison diagram of the four suppliers under different parameter value, exhibited in Figure 3. As it can be seen, the score value decreases with the increase in the θ for the same supplier, but the ranking outcome of four suppliers remains unchanged.

### 6.3. Comparison with Existing Methods

In this section, the results achieved by the proposed SC-IVPFHWAG and SC-IVPFOHWAG operators are compared with existing methods to verify their effectiveness and superiority. To illustrate the advantage of incorporating experts’ confidence with evaluation information, we adapt the methods proposed by [11] and compare them with our presented methods in this paper. Moreover, to express the superiority of the presented hybrid weighted methods in this paper, we choose the aggregation methods proposed in [49] as the comparison objects. Furthermore, by applying to the above case of green recycling suppliers of shared bicycles, the aggregation results of these methods are shown in detail in Table 7.

The results in Table 7 indicate that all the aggregation operators consistently consider supplier $A_{2}$ as the optimal option, and regard supplier $A_{1}$ as one with poor overall performance among the four suppliers except for IVPFWG and SC-IVPFWG operators. The ranking lists obtained by proposed aggregation methods remain completely identical with SC-IVPFOWA and SC-IVPFOWG operators, that is $A_{2}≻A_{3}≻A_{4}≻A_{1}$, which express slight divergence with other four aggregation operators, but all of them exert the same judgment on the optimal supplier in terms of comprehensive recycling performance. The results further verify the validity and effectiveness of the proposed methods in this paper. Through the above comparison, the reasons for the differences in ranking order are summarized.

(1) The outcomes acquired by IVPFWA and IVPFWG operators are not only different from most of the aggregation operators, but also the results obtained by the two aggregation operators are not completely consistent. The reason is that the former tends to the weighted average perspective, emphasizing group influence, while the latter tends to geometric average perspectives, emphasizing individual characteristics. The fundamental reason for the divergence between the two aggregation operators with other aggregation operators is that IVPFWA and IVPFWG operators ignore the degree of self-confidence of experts on their assessment value but assume that experts have a full grasp of their assessment information, that is, the self-confidence level of experts is 1, which is obviously not in accordance with the actual evaluation situation. In comparison, the aggregation methods proposed in this paper not only integrate the self-confidence level of experts but also effectively combine the aggregation ideas of weighted average and geometric average, making the results more convincing.
(2) Although the SC-IVPFWA and SC-IVPFWG operators overcome the shortcomings of the methods in [11], the results obtained by these two aggregation operators are obviously different, only possessing the identical judgment for the optimal supplier and completely inconsistent in other sorting orders. That is, the ranking results obtained by the weighted average aggregation and geometric average aggregation methods produce divergence. The main reason is that the weighted average aggregation and geometric average aggregation methods exhibit different aggregation focuses. While the ranking of the suppliers obtained by the proposed SC-IVPFHWAG and SC-IVPFOHWAG aggregation methods remain completely consistent, the reason is that they combine the characteristics of the two kinds of aggregation methods, not only retaining the individual influence but also emphasizing the group function; the information aggregation process is more reasonable.

Moreover, the proposed operators can choose suitable parameter values according to the decision-making requirements, making them more flexible in form than the above-mentioned weighted average and geometric operators. In general, the aggregation methods introduced in this paper not only consider the expert’s mastery of various attributes and fulfill the characteristics of actual cases, the obtained results are more rational and reliable both theoretically and practically, the final decision results are also greatly referential.

## 7. Conclusions

Considering the expert’s confidence with the evaluation of various attributes, this study combines the self-confidence level of experts with weighted average and geometric average aggregation methods and presents a novel hybrid aggregation approach based on SC-IVPFHWAG and SC-IVPFOHWAG operators. Some special expressions of the proposed aggregation operators are discussed under the specific values of parameter θ. To assess objectively and reasonably the recycling capacity of four green suppliers of shared bicycles, an evaluation index system for the selection of the shared bicycle recycling suppliers based on three traditional dimensions and the characteristic of shared bicycle recycling operations is constructed. Then, a novel hybrid MADM approach integrating the presented operators is applied for the green recycling supplier selection, which further demonstrates the applicability and efficiency of this approach. We also develop a group of experiments to study the influence of the parameter θ on the comprehensive ranking outcome and carry out comparisons with the existing approaches to prove the superiority and feasibility of this approach. All in all, the proposed approach possesses strong applicability to handle complex MADM problems.

The proposed operator can be easily extended to other extensions of fuzzy sets, such as Fermatean fuzzy sets [28], probabilistic hesitant fuzzy sets [24], etc. Our future work may consider extending the proposed method to other application fields, such as pattern recognition, financial investment, medical diagnosis, and so on.

## Figures and Tables

**Table 1:** Evaluation index system of green recycling suppliers of shared bicycles.

Dimension	Criterion	Literature
Economy ($C_{1}$)	Financial ability ($H_{1}$)	[1]
	Cost control ability ($H_{2}$)	[51,52,53,54,55,56,57,58,59,60,61,62,63]
	Quality assurance ($H_{3}$)	[54]
Environment ($C_{2}$)	Green image ($H_{4}$)	[5,50,55,56,57]
	Pollution and emission ($H_{5}$)	[1,58,59,60,61]
	Recovery capability ($H_{6}$)	[1,62,63]
Society ($C_{3}$)	Search ability ($H_{7}$)	[1,2]
	Cooperation with government ($H_{8}$)	[20,52,53]
	Publicity and education ($H_{9}$)	[20,52,53]

**Table 2:** IVPF decision matrix.

	$A_{1}$	$A_{2}$	$A_{3}$	$A_{4}$
$H_{1}$	$\langle \left[0.1,0.7\right],\left[0.5,0.7\right]\rangle$	$\langle \left[0.2,0.6\right],\left[0.7,0.8\right]\rangle$	$\langle \left[0.4,0.5,\left[0.6,0.7\right]\right]\rangle$	$\langle \left[0.1,0.4\right],\left[0.2,0.5\right]\rangle$
$H_{2}$	$\langle \left[0.5,0.6\right],\left[0.3,0.7\right]\rangle$	$\langle \left[0.3,0.5\right],\left[0.4,0.7\right]\rangle$	$\langle \left[0.3,0.7\right],\left[0.2,0.6\right]\rangle$	$\langle \left[0.2,0.4\right],\left[0.3,0.8\right]\rangle$
$H_{3}$	$\langle \left[0.2,0.4\right],\left[0.3,0.8\right]\rangle$	$\langle \left[0.7,0.8\right],\left[0.3,0.5\right]\rangle$	$\langle \left[0.1,0.6\right],\left[0.4,0.5\right]\rangle$	$\langle \left[0.4,0.6\right],\left[0.5,0.8\right]\rangle$
$H_{4}$	$\langle \left[0.4,,0.6\right],\left[0.5,0.7\right]\rangle$	$\langle \left[0.5,0.9\right],\left[0.3,0.4\right]\rangle$	$\langle \left[0.6,0.7\right],\left[0.3,0.7\right]\rangle$	$\langle \left[0.7,0.8\right],\left[0.1,0.4\right]\rangle$
$H_{5}$	$\langle \left[0.5,0.7\right],\left[0.4,0.3\right]\rangle$	$\langle \left[0.5,,0.6\right],\left[0.2,0.7\right]\rangle$	$\langle \left[0.4,0.7\right],\left[0.2,0.4\right]\rangle$	$\langle \left[0.6,0.8\right],\left[0.5,0.6\right]\rangle$
$H_{6}$	$\langle \left[0.4,0.7\right],\left[0.4,0.5\right]\rangle$	$\langle \left[0.5,0.6\right],\left[0.5,0.7\right]\rangle$	$\langle \left[0.5,0.7\right],\left[0.2,0.3\right]\rangle$	$\langle \left[0.1,0.5\right],\left[0.2,0.5\right]\rangle$
$H_{7}$	$\langle \left[0.3,0.7\right],\left[0.5,0.7\right]\rangle$	$\langle \left[0.8,0.9\right],\left[0.3,0.4\right]\rangle$	$\langle \left[0.7,0.8\right],\left[0.5,0.6\right]\rangle$	$\langle \left[0.3,0.5\right],\left[0.6,0.7\right]\rangle$
$H_{8}$	$\langle \left[0.7,0.8\right],\left[0.5,0.6\right]\rangle$	$\langle \left[0.5,0.8\right],\left[0.3,0.5\right]\rangle$	$\langle \left[0.3,0.4\right],\left[0.4,0.5\right]\rangle$	$\langle \left[0.5,0.6\right],\left[0.7,0.8\right]\rangle$
$H_{9}$	$\langle \left[0.4,0.6\right],\left[0.3,0.5\right]\rangle$	$\langle \left[0.5,0.8\right],\left[0.2,0.3\right]\rangle$	$\langle \left[0.4,0.7\right],\left[0.4,0.6\right]\rangle$	$\langle \left[0.5,0.8\right],\left[0.3,0.4\right]\rangle$

**Table 3:** Supplier aggregation results.

	SC-IVPFHWAG	SC-IVPFOHWAG
$A_{1}$	$\langle \left[0.4006,0.6289\right],\left[0.4760,0.6559\right]\rangle$	$\langle \left[0.3980,0.6334\right],\left[0.4714,0.6493\right]\rangle$
$A_{2}$	$\langle \left[0.5255,0.7304\right],\left[0.4375,0.5869\right]\rangle$	$\langle \left[0.5536,0.7384\right],\left[0.4366,0.5886\right]\rangle$
$A_{3}$	$\langle \left[0.4290,0.6335\right],\left[0.4554,0.6040\right]\rangle$	$\langle \left[0.4697,0.6434\right],\left[0.4359,0.5803\right]\rangle$
$A_{4}$	$\langle \left[0.4181,0.6259\right],\left[0.4301,0.6321\right]\rangle$	$\langle \left[0.4324,0.6274\right],\left[0.4362,0.6458\right]\rangle$

**Table 4:** Ranking results with various aggregation operators.

Aggregation Operator	Score Value	Ranking Result
SC−IPFHWAG	$\begin{array}{l} S_{A_{1}}=-0.0504\\ S_{A_{2}}=0.1368\\ S_{A_{3}}=0.0066\\ S_{A_{4}}=-0.0090 \end{array}$	$A_{2}≻A_{3}≻A_{4}≻A_{1}$
SC−IPFOHWAG	$\begin{array}{l} S_{A_{1}}=-0.0421\\ S_{A_{2}}=0.1573\\ S_{A_{3}}=0.0539\\ S_{A_{4}}=-0.0133 \end{array}$	$A_{2}≻A_{3}≻A_{4}≻A_{1}$

**Table 5:** Variation in SC-IVPFHWAG with θ.

θ	Ranking of Score Values	Preference Order
0	$S_{A_{2}}>S_{A_{3}}>S_{A_{1}}>S_{A_{4}}$	$A_{2}≻A_{3}≻A_{1}≻A_{4}$
0.05	$S_{A_{2}}>S_{A_{3}}>S_{A_{1}}>S_{A_{4}}$	$A_{2}≻A_{3}≻A_{1}≻A_{4}$
0.10	$S_{A_{2}}>S_{A_{3}}>S_{A_{4}}>S_{A_{1}}$	$A_{2}≻A_{3}≻A_{4}≻A_{1}$
0.15	$S_{A_{2}}>S_{A_{3}}>S_{A_{4}}>S_{A_{1}}$	$A_{2}≻A_{3}≻A_{4}≻A_{1}$
0.20	$S_{A_{2}}>S_{A_{3}}>S_{A_{4}}>S_{A_{1}}$	$A_{2}≻A_{3}≻A_{4}≻A_{1}$
0.25	$S_{A_{2}}>S_{A_{3}}>S_{A_{4}}>S_{A_{1}}$	$A_{2}≻A_{3}≻A_{4}≻A_{1}$
0.30	$S_{A_{2}}>S_{A_{3}}>S_{A_{4}}>S_{A_{1}}$	$A_{2}≻A_{3}≻A_{4}≻A_{1}$
0.35	$S_{A_{2}}>S_{A_{3}}>S_{A_{4}}>S_{A_{1}}$	$A_{2}≻A_{3}≻A_{4}≻A_{1}$
0.40	$S_{A_{2}}>S_{A_{3}}>S_{A_{4}}>S_{A_{1}}$	$A_{2}≻A_{3}≻A_{4}≻A_{1}$
0.45	$S_{A_{2}}>S_{A_{3}}>S_{A_{4}}>S_{A_{1}}$	$A_{2}≻A_{3}≻A_{4}≻A_{1}$
0.50	$S_{A_{2}}>S_{A_{3}}>S_{A_{4}}>S_{A_{1}}$	$A_{2}≻A_{3}≻A_{4}≻A_{1}$
0.55	$S_{A_{2}}>S_{A_{3}}>S_{A_{4}}>S_{A_{1}}$	$A_{2}≻A_{3}≻A_{4}≻A_{1}$
0.60	$S_{A_{2}}>S_{A_{3}}>S_{A_{4}}>S_{A_{1}}$	$A_{2}≻A_{3}≻A_{4}≻A_{1}$
0.65	$S_{A_{2}}>S_{A_{3}}>S_{A_{4}}>S_{A_{1}}$	$A_{2}≻A_{3}≻A_{4}≻A_{1}$
0.70	$S_{A_{2}}>S_{A_{3}}>S_{A_{4}}>S_{A_{1}}$	$A_{2}≻A_{3}≻A_{4}≻A_{1}$
0.75	$S_{A_{2}}>S_{A_{4}}>S_{A_{3}}>S_{A_{1}}$	$A_{2}≻A_{4}≻A_{3}≻A_{1}$
0.80	$S_{A_{2}}>S_{A_{4}}>S_{A_{3}}>S_{A_{1}}$	$A_{2}≻A_{4}≻A_{3}≻A_{1}$
0.85	$S_{A_{2}}>S_{A_{4}}>S_{A_{3}}>S_{A_{1}}$	$A_{2}≻A_{4}≻A_{3}≻A_{1}$
0.90	$S_{A_{2}}>S_{A_{4}}>S_{A_{3}}>S_{A_{1}}$	$A_{2}≻A_{4}≻A_{3}≻A_{1}$
0.95	$S_{A_{2}}>S_{A_{4}}>S_{A_{3}}>S_{A_{1}}$	$A_{2}≻A_{4}≻A_{3}≻A_{1}$
1	$S_{A_{2}}>S_{A_{4}}>S_{A_{3}}>S_{A_{1}}$	$A_{2}≻A_{4}≻A_{3}≻A_{1}$

**Table 6:** Variation in SC-IVPFOHWAG with θ.

θ	Ranking of Score Values	Preference Order
0	$S_{A_{2}}>S_{A_{3}}>S_{A_{4}}>S_{A_{1}}$	$A_{2}≻A_{3}≻A_{4}≻A_{1}$
0.05	$S_{A_{2}}>S_{A_{3}}>S_{A_{4}}>S_{A_{1}}$	$A_{2}≻A_{3}≻A_{4}≻A_{1}$
0.10	$S_{A_{2}}>S_{A_{3}}>S_{A_{4}}>S_{A_{1}}$	$A_{2}≻A_{3}≻A_{4}≻A_{1}$
0.15	$S_{A_{2}}>S_{A_{3}}>S_{A_{4}}>S_{A_{1}}$	$A_{2}≻A_{3}≻A_{4}≻A_{1}$
0.20	$S_{A_{2}}>S_{A_{3}}>S_{A_{4}}>S_{A_{1}}$	$A_{2}≻A_{3}≻A_{4}≻A_{1}$
0.25	$S_{A_{2}}>S_{A_{3}}>S_{A_{4}}>S_{A_{1}}$	$A_{2}≻A_{3}≻A_{4}≻A_{1}$
0.30	$S_{A_{2}}>S_{A_{3}}>S_{A_{4}}>S_{A_{1}}$	$A_{2}≻A_{3}≻A_{4}≻A_{1}$
0.35	$S_{A_{2}}>S_{A_{3}}>S_{A_{4}}>S_{A_{1}}$	$A_{2}≻A_{3}≻A_{4}≻A_{1}$
0.40	$S_{A_{2}}>S_{A_{3}}>S_{A_{4}}>S_{A_{1}}$	$A_{2}≻A_{3}≻A_{4}≻A_{1}$
0.45	$S_{A_{2}}>S_{A_{3}}>S_{A_{4}}>S_{A_{1}}$	$A_{2}≻A_{3}≻A_{4}≻A_{1}$
0.50	$S_{A_{2}}>S_{A_{3}}>S_{A_{4}}>S_{A_{1}}$	$A_{2}≻A_{3}≻A_{4}≻A_{1}$
0.55	$S_{A_{2}}>S_{A_{3}}>S_{A_{4}}>S_{A_{1}}$	$A_{2}≻A_{3}≻A_{4}≻A_{1}$
0.60	$S_{A_{2}}>S_{A_{3}}>S_{A_{4}}>S_{A_{1}}$	$A_{2}≻A_{3}≻A_{4}≻A_{1}$
0.65	$S_{A_{2}}>S_{A_{3}}>S_{A_{4}}>S_{A_{1}}$	$A_{2}≻A_{3}≻A_{4}≻A_{1}$
0.70	$S_{A_{2}}>S_{A_{3}}>S_{A_{4}}>S_{A_{1}}$	$A_{2}≻A_{3}≻A_{4}≻A_{1}$
0.75	$S_{A_{2}}>S_{A_{3}}>S_{A_{4}}>S_{A_{1}}$	$A_{2}≻A_{3}≻A_{4}≻A_{1}$
0.80	$S_{A_{2}}>S_{A_{3}}>S_{A_{4}}>S_{A_{1}}$	$A_{2}≻A_{3}≻A_{4}≻A_{1}$
0.85	$S_{A_{2}}>S_{A_{3}}>S_{A_{4}}>S_{A_{1}}$	$A_{2}≻A_{3}≻A_{4}≻A_{1}$
0.90	$S_{A_{2}}>S_{A_{3}}>S_{A_{4}}>S_{A_{1}}$	$A_{2}≻A_{3}≻A_{4}≻A_{1}$
0.95	$S_{A_{2}}>S_{A_{3}}>S_{A_{4}}>S_{A_{1}}$	$A_{2}≻A_{3}≻A_{4}≻A_{1}$
1	$S_{A_{2}}>S_{A_{3}}>S_{A_{4}}>S_{A_{1}}$	$A_{2}≻A_{3}≻A_{4}≻A_{1}$

**Table 7:** Results rendered by various aggregation methods.

Operator	Aggregation Value	Score Value	Ranking Result
IVPFWA [11]	$\begin{array}{l} \psi_{A_{1}}=\langle \left[0.4403,0.6605\right],\left[0.3982,0.6059\right]\rangle \;\\ \psi_{A_{2}}=\langle \left[0.5284,0.7698\right],\left[0.3317,0.5149\right]\rangle \\ \psi_{A_{3}}=\langle \left[0.4317,0.6446\right],\left[0.3524,0.5476\right]\rangle \\ \psi_{A_{4}}=\langle \left[0.4556,0.6549\right],\left[0.3114,0.5749\right]\rangle \; \end{array}$	$\begin{array}{l} S_{A_{1}}=0.0522\\ S_{A_{2}}=0.2483\\ S_{A_{3}}=0.0889\\ S_{A_{4}}=0.1045 \end{array}$	$A_{2}≻A_{4}≻A_{3}≻A_{1}$
IVPFWG [11]	$\begin{array}{l} \psi_{A_{1}}=\langle \left[0.3271,0.6264\right],\left[0.4231,0.6521\right]\rangle \;\\ \psi_{A_{2}}=\langle \left[0.4473,0.7185\right],\left[0.4175,0.5961\right]\rangle \\ \psi_{A_{3}}=\langle \left[0.3456,0.6080\right],\left[0.4062,0.5843\right]\rangle \\ \psi_{A_{4}}=\langle \left[0.3159,0.5869\right],\left[0.4378,0.6524\right]\rangle \; \end{array}$	$\begin{array}{l} S_{A_{1}}=-0.0524\\ S_{A_{2}}=0.0923\\ S_{A_{3}}=-0.0086\\ S_{A_{4}}=-0.0865 \end{array}$	$A_{2}≻A_{3}≻A_{1}≻A_{4}$
SC−IVPFWA [49]	$\begin{array}{l} \psi_{A_{1}}=\langle \left[0.3214,0.5266\right],\left[0.5749,0.7413\right]\rangle \;\\ \psi_{A_{2}}=\langle \left[0.4363,0.6451\right],\left[0.5175,0.6679\right]\rangle \\ \psi_{A_{3}}=\langle \left[0.3458,0.5311\right],\left[0.5464,0.6967\right]\rangle \\ \psi_{A_{4}}=\langle \left[0.3502,0.5343\right],\left[0.5026,0.7130\right]\rangle \; \end{array}$	$\begin{array}{l} S_{A_{1}}=-0.2497\\ S_{A_{2}}=-0.0537\\ S_{A_{3}}=-0.1911\\ S_{A_{4}}=-0.1765 \end{array}$	$A_{2}≻A_{4}≻A_{3}≻A_{1}$
SC−IVPFWG [49]	$\begin{array}{l} \psi_{A_{1}}=\langle \left[0.4994,0.7510\right],\left[0.3265,0.5287\right]\rangle \\ \psi_{A_{2}}=\langle \left[0.6330,0.8268\right],\left[0.3273,0.4735\right]\rangle \\ \psi_{A_{3}}=\langle \left[0.5323,0.7558\right],\left[0.3231,0.4648\right]\rangle \\ \psi_{A_{4}}=\langle \left[0.4993,0.7332\right],\left[0.3335,0.5163\right]\rangle \; \end{array}$	$\begin{array}{l} S_{A_{1}}=0.2137\\ S_{A_{2}}=0.3765\\ S_{A_{3}}=0.2671\\ S_{A_{4}}=0.2045 \end{array}$	$A_{2}≻A_{3}≻A_{1}≻A_{4}$
SC−IVPFHWAG	$\begin{array}{l} \psi_{A_{1}}=\langle \left[0.4006,0.6289\right],\left[0.4760,0.6559\right]\rangle \;\\ \psi_{A_{2}}=\langle \left[0.5255,0.7304\right],\left[0.4375,0.5869\right]\rangle \\ \psi_{A_{3}}=\langle \left[0.4290,0.6335\right],\left[0.4554,0.6040\right]\rangle \\ \psi_{A_{4}}=\langle \left[0.4181,0.6259\right],\left[0.4301,0.6321\right]\rangle \; \end{array}$	$\begin{array}{l} S_{A_{1}}=-0.0504\\ S_{A_{2}}=0.1368\\ S_{A_{3}}=0.0066\\ S_{A_{4}}=-0.0090 \end{array}$	$A_{2}≻A_{3}≻A_{4}≻A_{1}$
SC−IVPFOWA [49]	$\begin{array}{l} \psi_{A_{1}}=\langle \left[0.3244,0.5379\right],\left[0.5669,0.7295\right]\rangle \\ \psi_{A_{2}}=\langle \left[0.4818,0.6636\right],\left[0.5122,0.6648\right]\rangle \\ \psi_{A_{3}}=\langle \left[0.3777,0.5433\right],\left[0.5211,0.6696\right]\rangle \\ \psi_{A_{4}}=\langle \left[0.3587,0.5282\right],\left[0.5139,0.7334\right]\rangle \; \end{array}$	$\begin{array}{l} S_{A_{1}}=-0.2295\\ S_{A_{2}}=-0.0159\\ S_{A_{3}}=-0.1410\\ S_{A_{4}}=-0.1972 \end{array}$	$A_{2}≻A_{3}≻A_{4}≻A_{1}$
SC−IVPFOWG [49]	$\begin{array}{l} \psi_{A_{1}}=\langle \left[0.4883,0.7460\right],\left[0.3294,0.5338\right]\rangle \\ \psi_{A_{2}}=\langle \left[0.6361,0.8216\right],\left[0.3345,0.4843\right]\rangle \\ \psi_{A_{3}}=\langle \left[0.5841,0.7618\right],\left[0.3153,0.4501\right]\rangle \\ \psi_{A_{4}}=\langle \left[0.5214,0.7453\right],\left[0.3303,0.5143\right]\rangle \end{array}$	$\begin{array}{l} S_{A_{1}}=0.2007\\ S_{A_{2}}=0.3666\\ S_{A_{3}}=0.3098\\ S_{A_{4}}=0.2268 \end{array}$	$A_{2}≻A_{3}≻A_{4}≻A_{1}$
SC−IVPFOHWAG	$\begin{array}{l} \psi_{A_{1}}=\langle \left[0.3980,0.6334\right],\left[0.4714,0.6493\right]\rangle \\ \psi_{A_{2}}=\langle \left[0.5536,0.7384\right],\left[0.4366,0.5886\right]\rangle \\ \psi_{A_{3}}=\langle \left[0.4697,0.6434\right],\left[0.4359,0.5803\right]\rangle \\ \psi_{A_{4}}=\langle \left[0.4324,0.6274\right],\left[0.4362,0.6458\right]\rangle \end{array}$	$\begin{array}{l} S_{A_{1}}=-0.0421\\ S_{A_{2}}=0.1537\\ S_{A_{3}}=0.0539\\ S_{A_{4}}=-0.0133 \end{array}$	$A_{2}≻A_{3}≻A_{4}≻A_{1}$

## Data Availability

Not applicable.
